# Supplementary figures and images for: Single-cell transcriptomic analysis reveals rich pituitary–Immune interactions under systemic inflammation
Source: PLoS Biol. 2023 Dec 18;21(12):e3002403. doi: 10.1371/journal.pbio.3002403 (PMC10727439; doi:10.1371/journal.pbio.3002403)

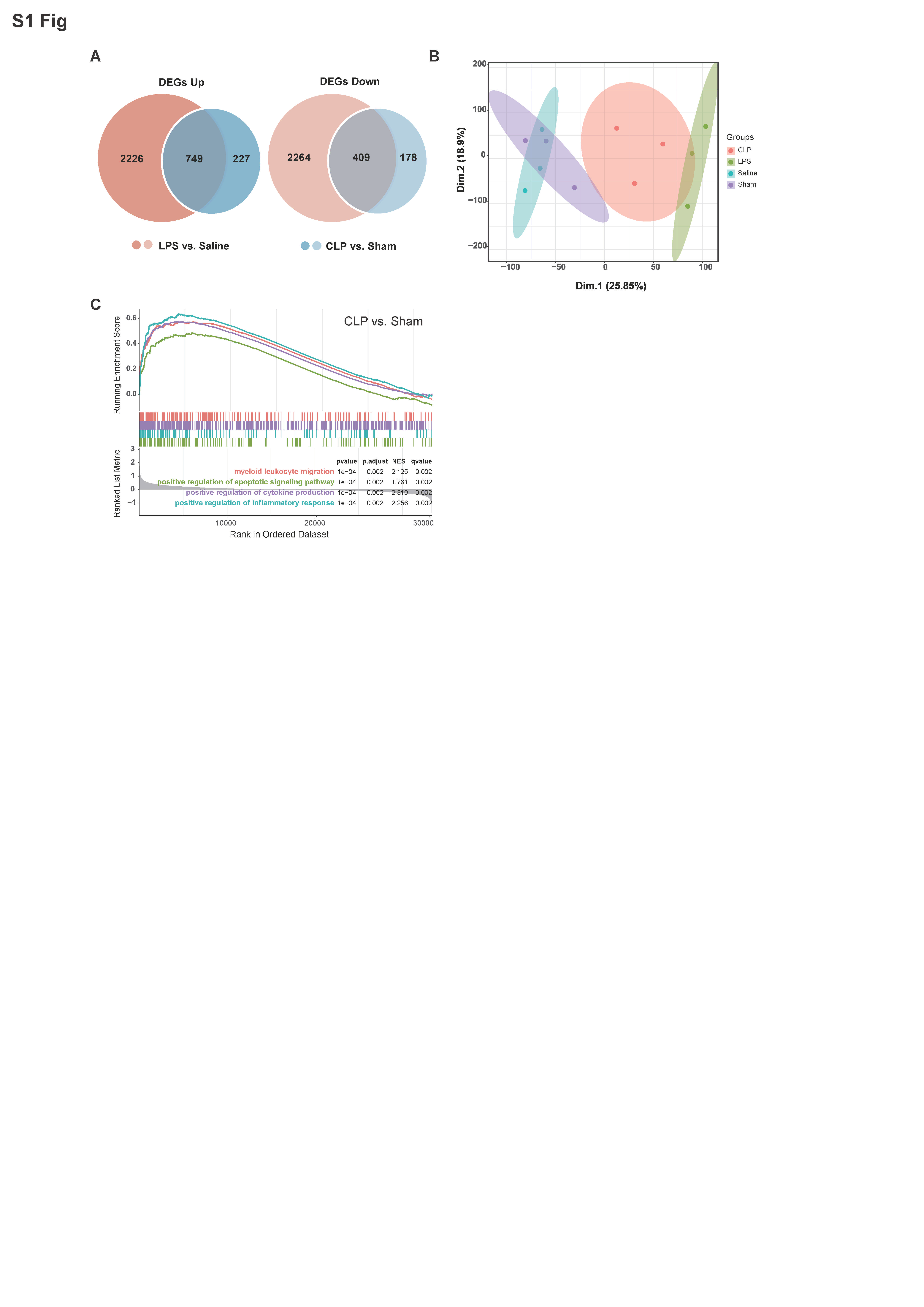

Supplement: S1 Fig — (A) Venn diagrams showing the intersection of up-regulated (left panel) and down-regulated (right panel) DEGs in the pituitary under medium-dose (5 mg/kg) LPS (i.p.) treatment and mid-grade CLP treatment for 6 h, and the control groups treated with saline (i.p.) or sham surgery. (B) PCA of the pituitary transcriptomes from mice subjected to LPS, CLP for 6 h, and the control groups as indicated in (A) (n = 3 replicates). (C) GSEA profiles showing significant enrichment of gene sets after CLP treatment in the pituitary. The transcriptome datasets used in (A) were used for GSEA analysis. The data underlying this figure can be found in S1 Table. CLP, cecal ligation and puncture; PCA, Principal component analysis; GSEA, Gene Set Enrichment Analysis. (TIFF) [file pbio.3002403.s001.tiff]

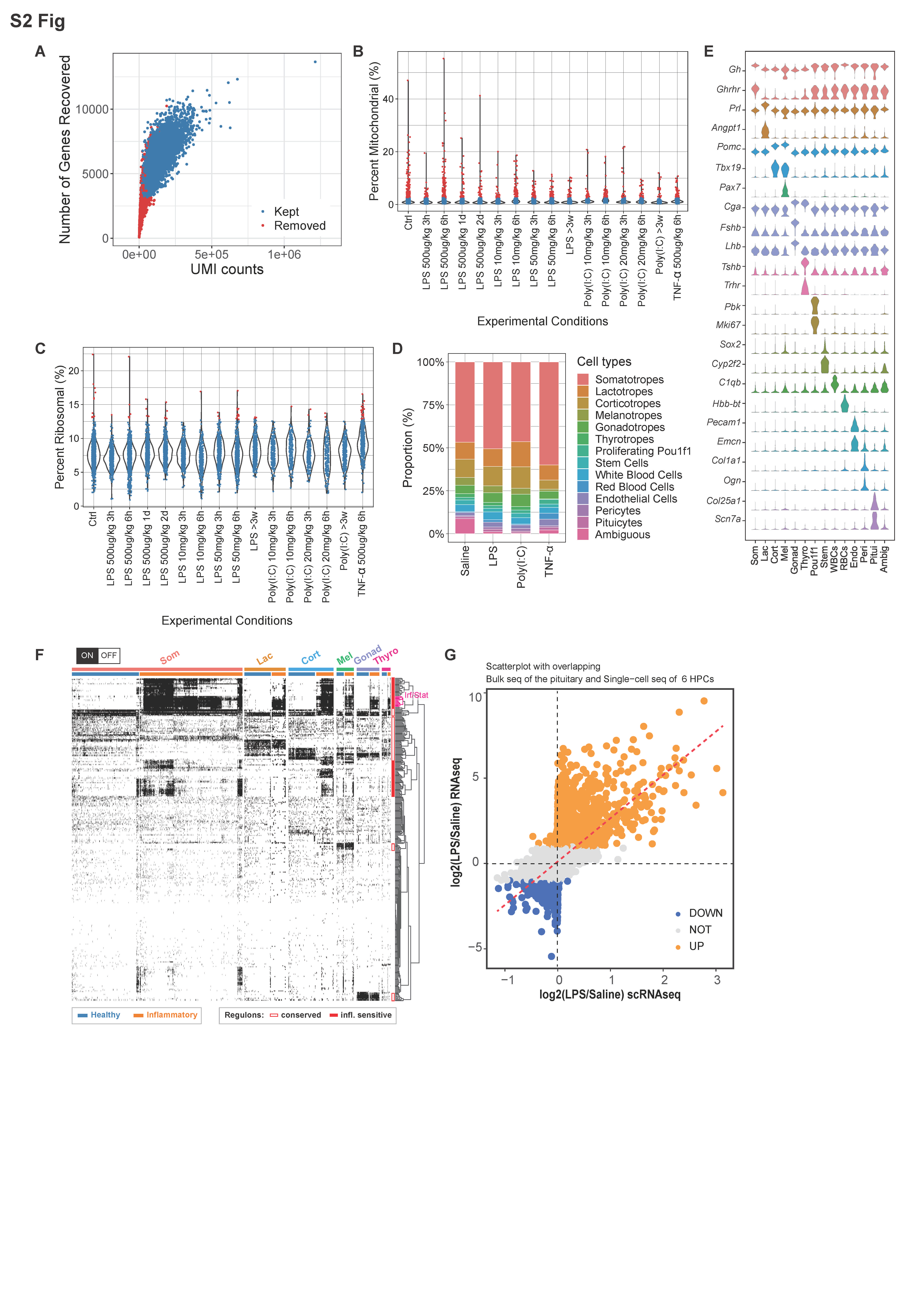

Supplement: S2 Fig — (A–C) Quality control metrics showing the number of genes recovered per cell and UMI counts recovered per cell (A), mitochondrial RNA fraction (B), and ribosomal RNA fraction (C). Dots marked in red are cells that failed to pass the QC metrics and were removed from subsequent analyses. (D) Cell type composition of the sequenced pituitary single cells. (E) Violin plots showing expression of canonical marker genes for identified pituitary cell types as indicated in (Fig 1E). (F) Heatmap showing the 356 binarized SCENIC regulon activity in HPCs with healthy and inflammatory cell states predicted in (Fig 1I). Columns are cells and rows are regulons. White: not activated; black: activated. (G) Scatterplots showing the correlation between scRNA-seq of 6 HPCs and bulk RNA-seq of the pituitary under LPS treatment. Genes up-regulated or down-regulated by more than 2-fold in bulk RNA-seq are indicated in orange and blue, respectively. Dashed line represents the fitting curve. The data underlying this figure can be found in S2 and S4 Tables. (TIFF) [file pbio.3002403.s002.tiff]

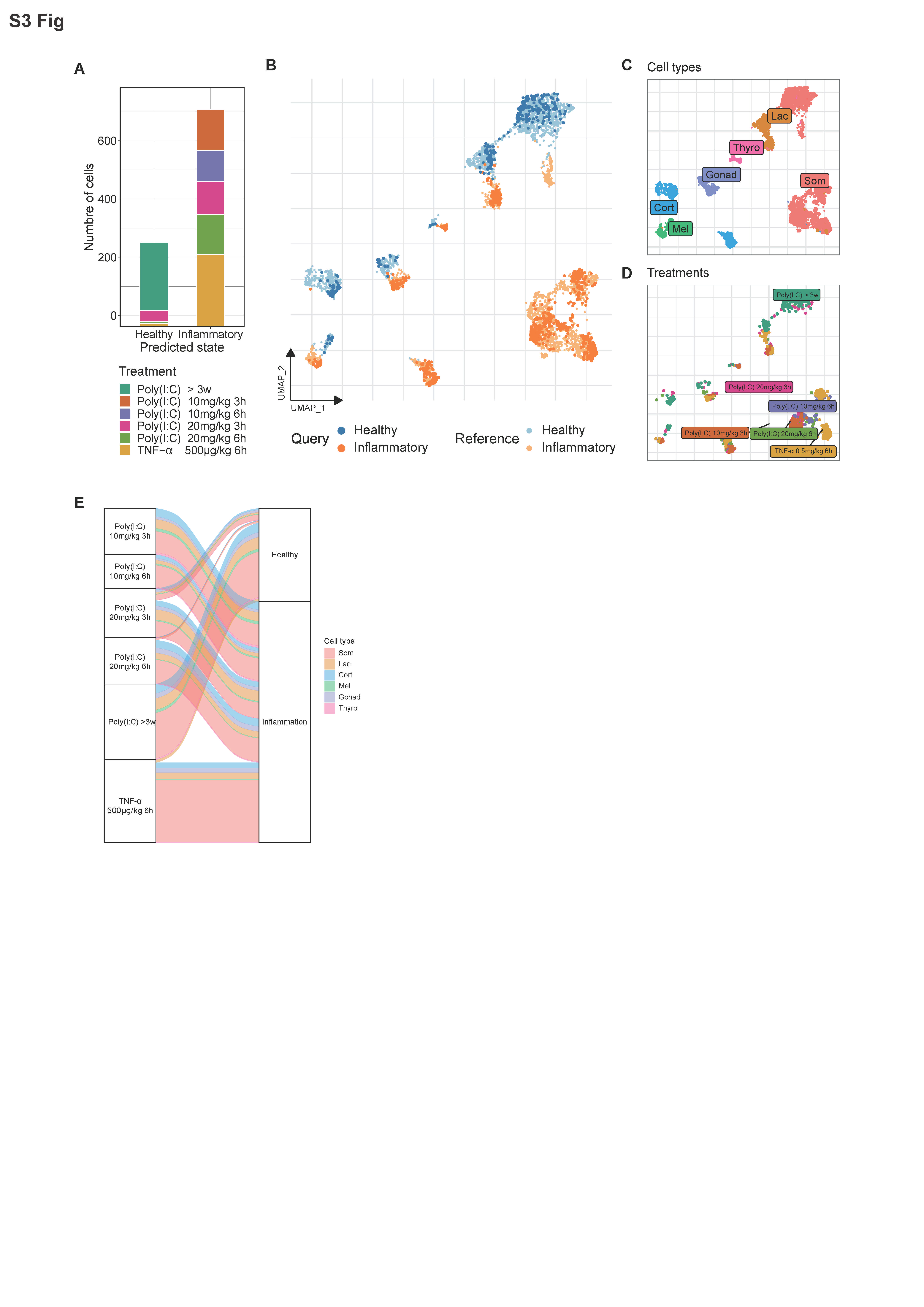

Supplement: S3 Fig — (A) Bar plot showing the predicted states of HPCs in Poly(I:C) and TNF-α groups. (B) Joint embedding of pituitary single cells using UMAP, with the query dataset [Poly(I:C) and TNF-α] projected onto the reference structure [Saline and LPS treatments]. (C and D) Visualization of all cell types (C) and treatments (D) in the query dataset, using the same UMAP embedding as in (B). (E) Alluvial plot showing the cell state distribution of 6 HPCs under Poly(I:C) and TNF-α treatment groups. HPCs, hormone-producing cells. (TIFF) [file pbio.3002403.s003.tiff]

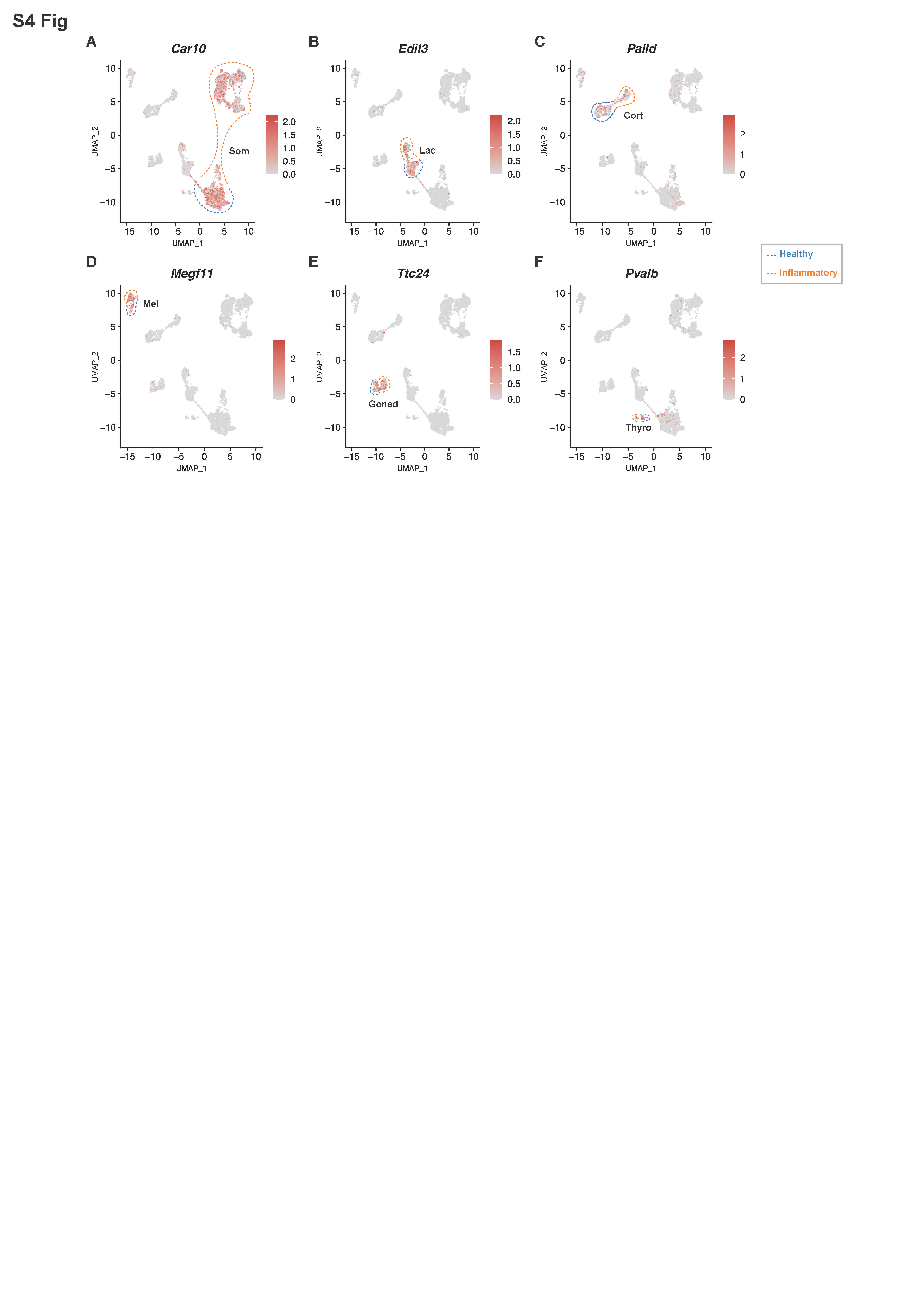

Supplement: S4 Fig — (A–F) UMAP plots showing conserved marker genes for pituitary HPCs. Car10 is used as a conserved marker for somatotropes (A), Edil3 for lactotropes (B), Palld for corticotropes (C), Megf11 for melanotropes (D), Ttc24 for gonadotropes (E), and Pvalb for thyrotropes (F). HPCs, hormone-producing cells. (TIFF) [file pbio.3002403.s004.tiff]

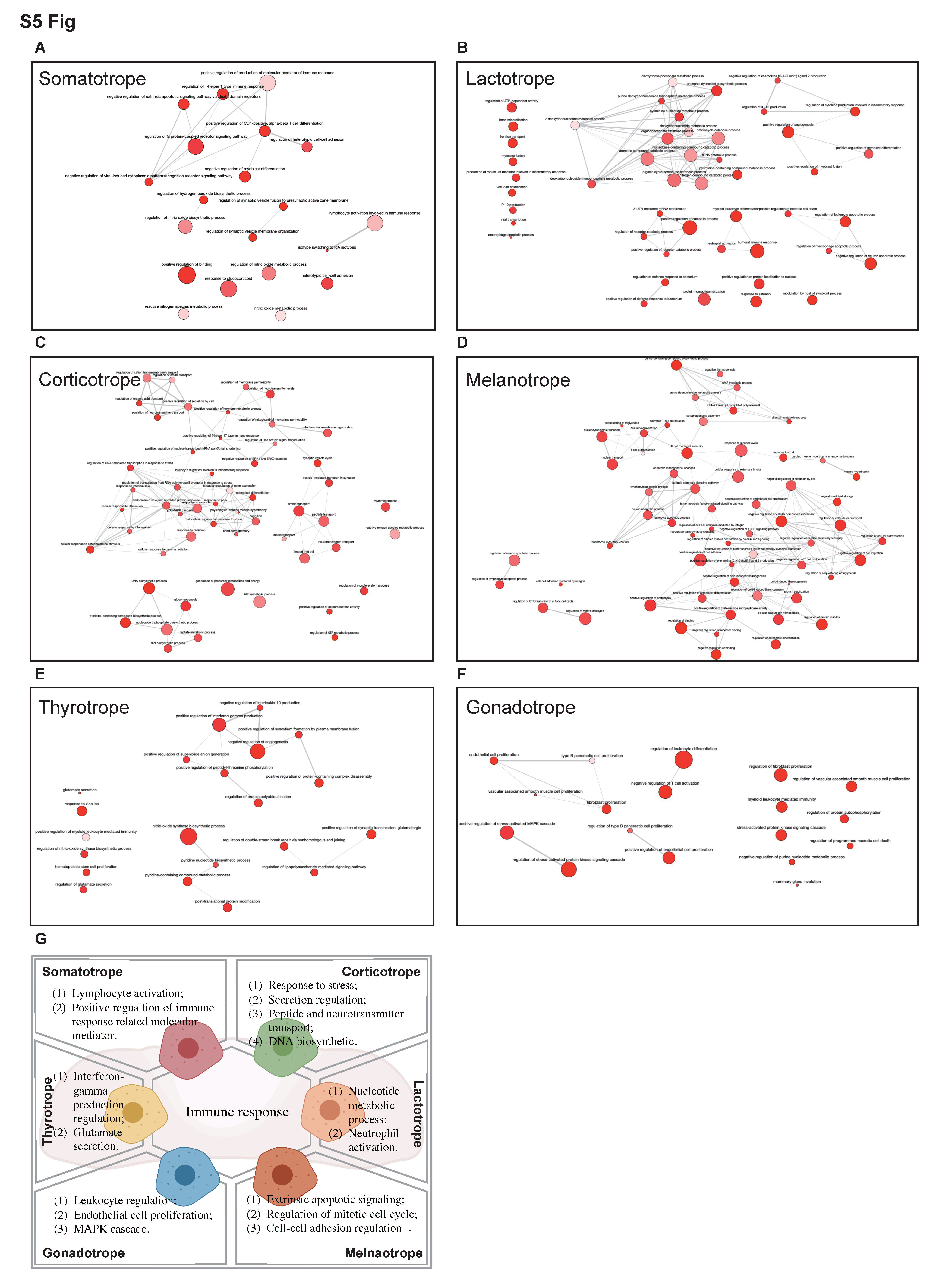

Supplement: S5 Fig — (A–F) REVIGO analysis showing the interactive graph of 6 HPCs under systemic inflammation. Bubble color indicates the p-value. Bubble size indicates the frequency of the GO term in the underlying GOA database. (G) Summary shared and unique cell signaling pathways in HPCs from (A–F). The representations were created with BioRender.com. REVIGO analysis URL (http://revigo.irb.hr). The data underlying this figure can be found in S9 Table. GOA, Gene Ontology Annotation. (TIFF) [file pbio.3002403.s005.tiff]

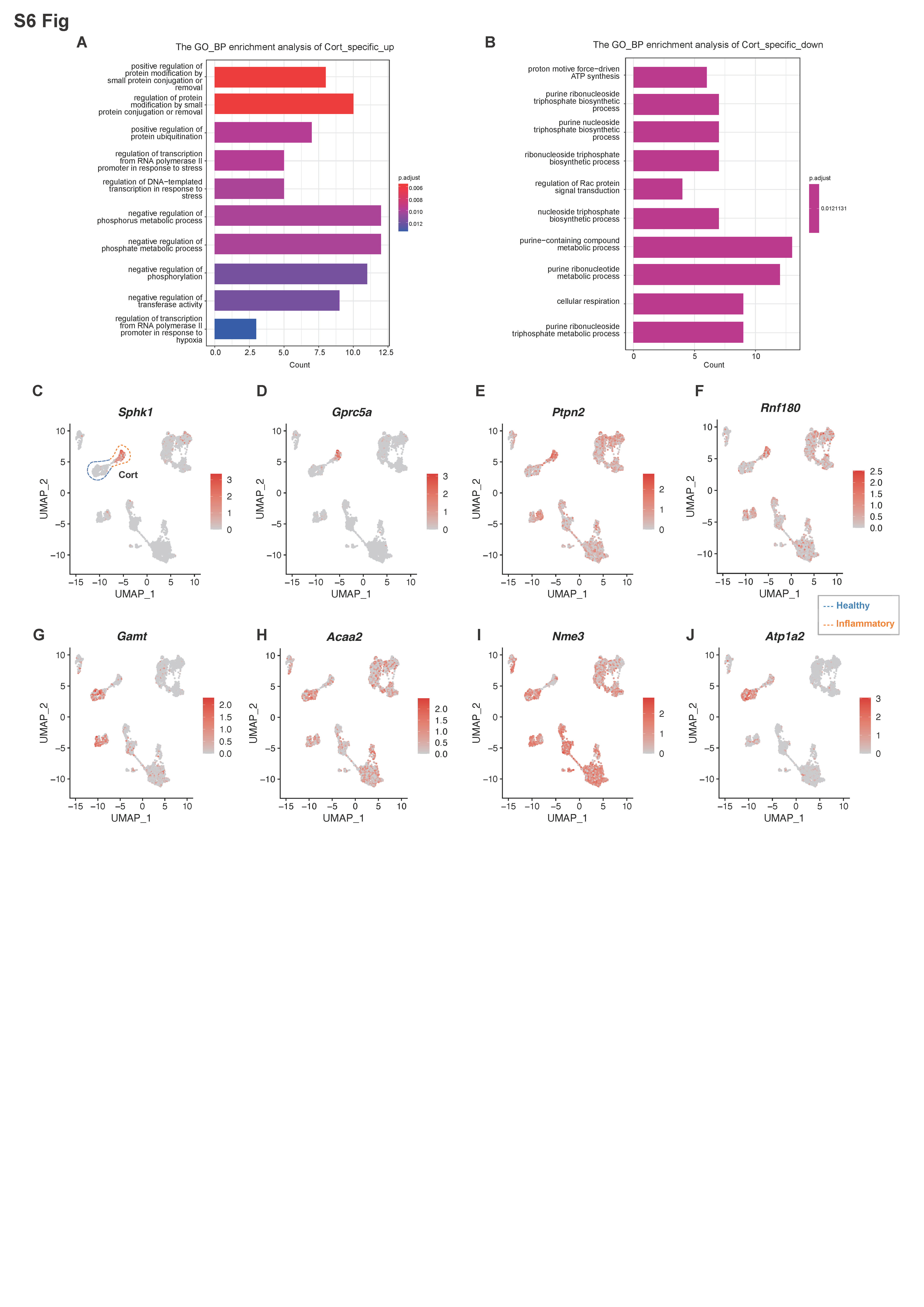

Supplement: S6 Fig — (A and B) GO analysis on unique up-regulated (A) and down-regulated (B) DEGs of corticotropes. (C–F) UMAP plots showing up-regulated DEGs in corticotropes. (G–J) UMAP plots showing down-regulated DEGs in corticotropes. The data underlying this figure can be found in S10 Table. (TIFF) [file pbio.3002403.s006.tiff]

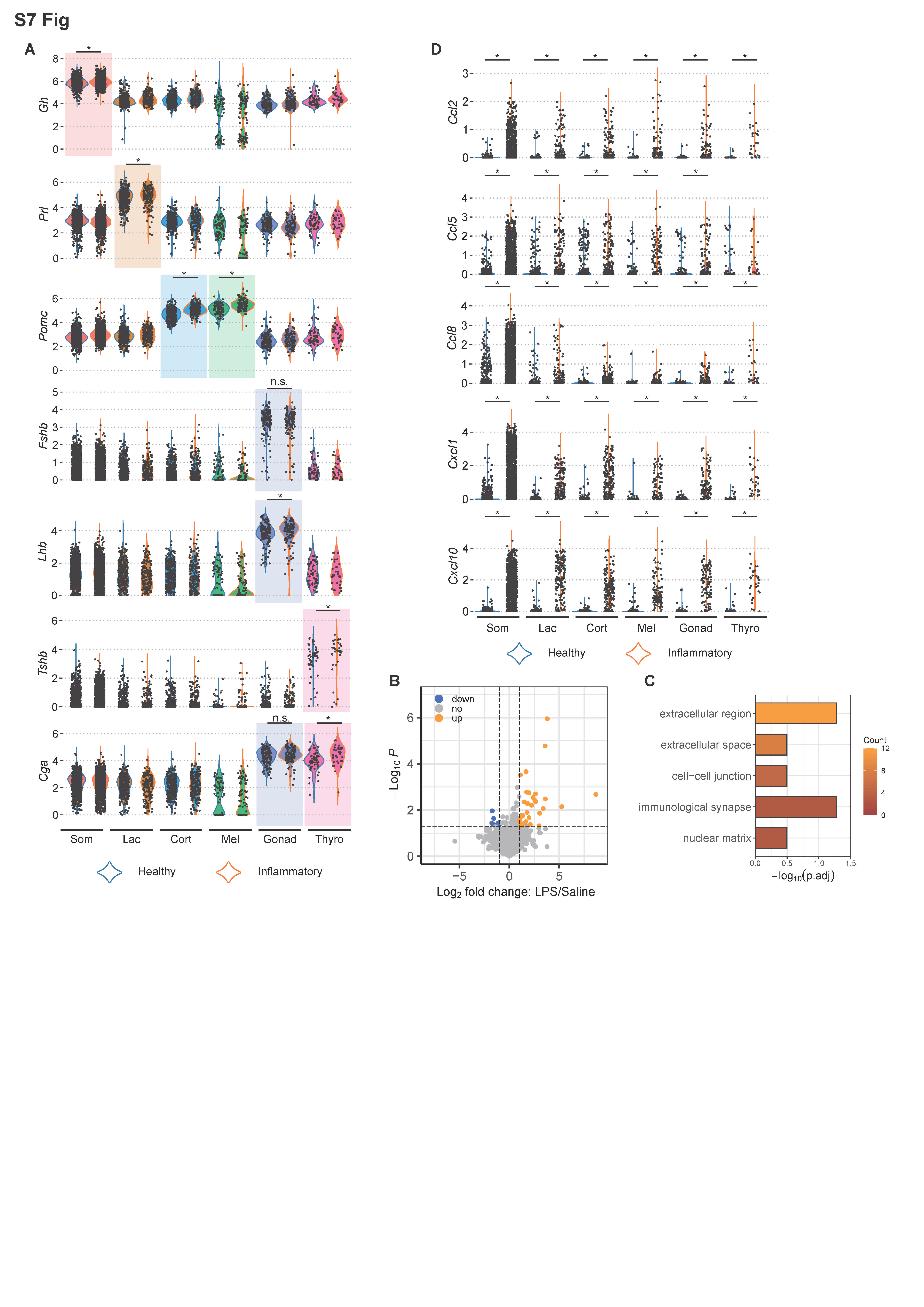

Supplement: S7 Fig — (A) Violin plots showing the expression of canonical HPCs marker genes under healthy or inflammatory states. Violin line color: cell states. (B) Volcano plot showing the pituitary proteome for differentially abundant proteins in control and LPS (50 mg/kg LPS for 6 h) groups. (C) GO analysis on up-regulated proteins from (B). (D) Violin plots showing the expression of myeloid migration-related chemokine genes under healthy or inflammatory states. Violin line color: cell states. The data underlying this figure can be found in S12 Table. HPCs, hormone-producing cells. (TIFF) [file pbio.3002403.s007.tiff]

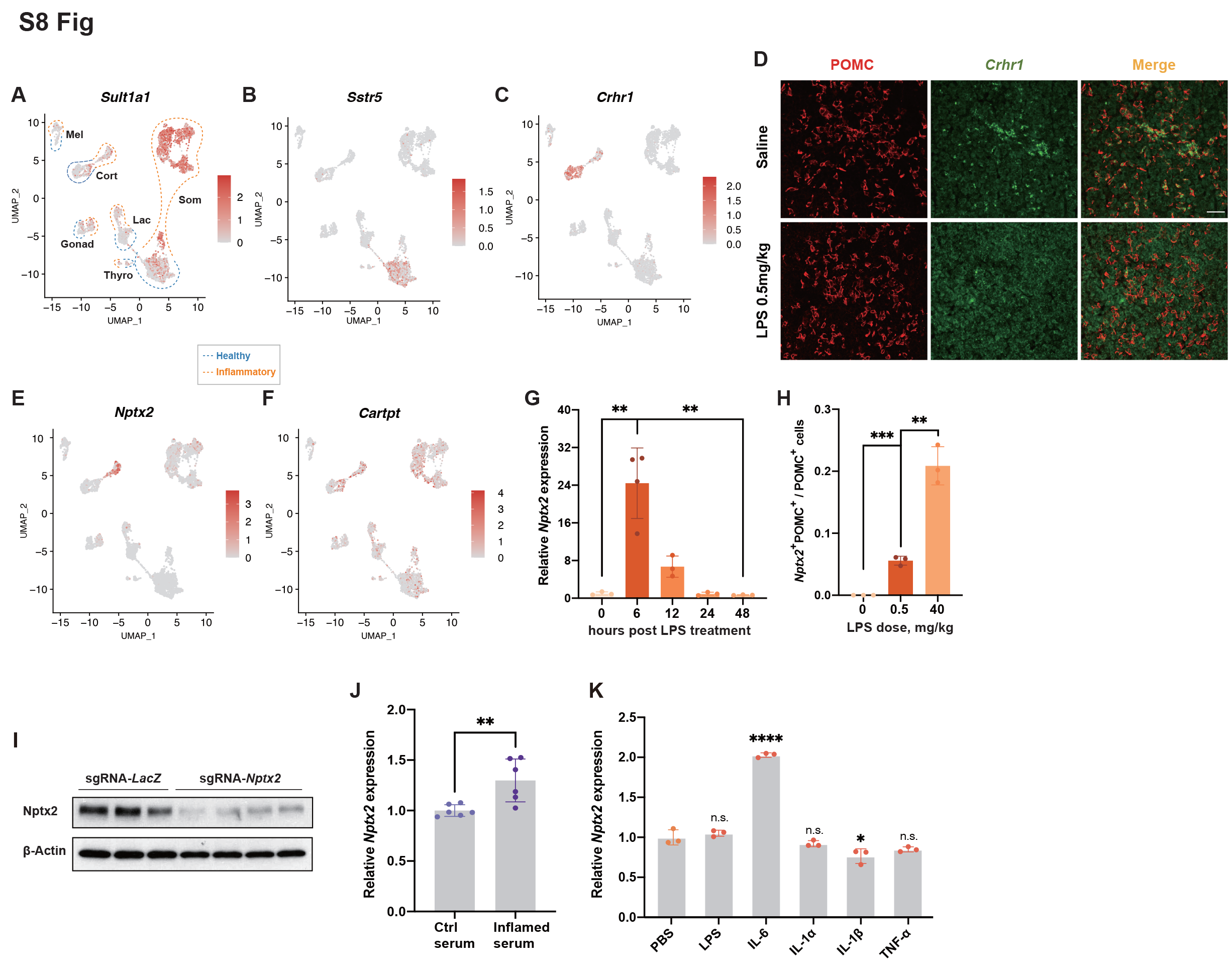

Supplement: S8 Fig — (A–C) UMAP plots showing DEGs identified from the scRNA-seq dataset. (D) Representative images showing ISH of Crhr1 RNA (green) and IF of POMC (red) in the pituitary from mice treated with saline or LPS for 6 h. Scale bar, 50 μm. (E and F) UMAP plots showing Nptx2 (E) and Cartpt (F) from the scRNA-seq dataset. (G) qPCR analysis of Nptx2 in the pituitary from mice treated with LPS (0.5 mg/kg) for different durations (n = 3–4 mice). (H) Histogram showing the statistic information of the percentage of Nptx2+ cells in POMC+ cells. (I) Immunoblot analysis of Nptx2 in the pituitary from LPS (0.5 mg/kg LPS exposure for 6 h)-challenged mice 3 weeks following the infusion of AAV-SaCas9-U6-sgRNA-Nptx2 or the control vectors into the pituitary (n = 3 or 4 mice per group). (J) qPCR analysis of Nptx2 in AtT-20 cells after treatment with control or inflamed serum for 6 h (n = 6 replicates). (K) qPCR analysis of Nptx2 in AtT-20 cells after treatment with PBS, 200 ng/mL LPS, or 100 ng/mL cytokines for 6 h (n = 3 replicates). All data represent mean with SD. Statistical significance was determined by two-tailed Student’s t test, *p < 0.05, **p < 0.01, ***p < 0.001, ****p < 0.0001. The data underlying this figure can be found in S1 Data. The original blot for this blot can be found in S1 Raw Images. ISH, in situ hybridization; IF, immunofluorescence. (TIF) [file pbio.3002403.s008.tif]

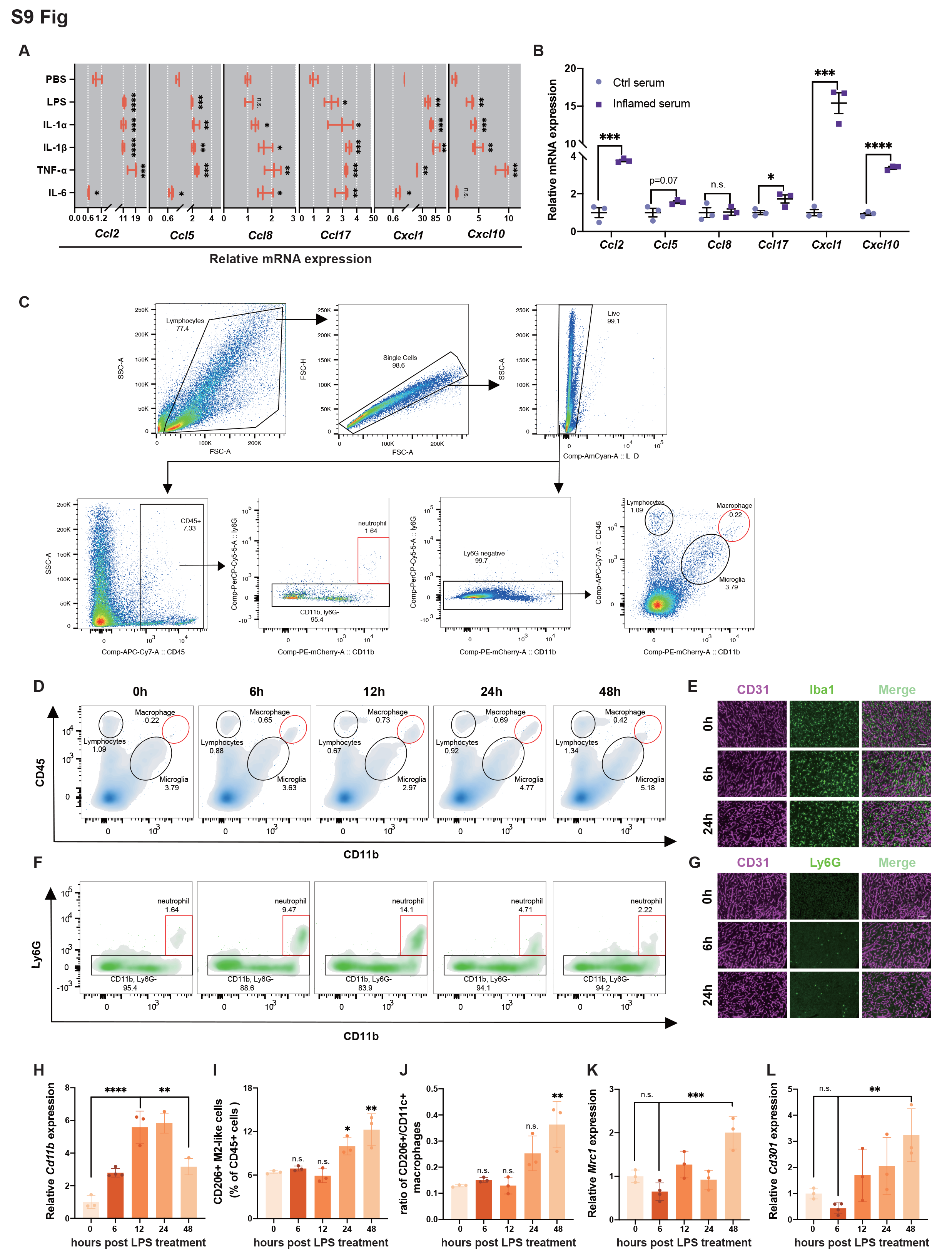

Supplement: S9 Fig — (A) qPCR analysis of several major chemokine-encoding genes in AtT-20 cells after treatment with PBS, 200 ng/mL LPS, or 100 ng/mL cytokines for 6 h (n = 3 replicates). (B) qPCR analysis of the chemokine-encoding genes in AtT-20 cells after treatment with control or inflamed serum for 6 h (n = 3 replicates). (C) FACS gating strategies for neutrophil and microglia/macrophage. (D) FACS analysis for macrophages (red circle) among Ly6G- cells in the pituitary from mice treated with LPS at different times. (E) Representative images showing IF of CD31 (purple, the marker of endothelial cell) and Iba1 (green) in the pituitary from mice treated with saline or LPS. Scale bar, 100 μm. (F) FACS analysis for neutrophils (red rectangle) among CD45+ cells in the pituitary from mice treated with LPS at different times. (G) Representative images showing IF of CD31 (purple) and Ly6G (green, the marker of neutrophil) in the pituitary from mice treated with saline or LPS. Scale bar, 100 μm. (H) qPCR analysis of the representative macrophage marker gene Cd11b in the pituitary from mice treated with LPS. (I and J) FACS analysis of CD206+ M2-like macrophages (I) and the ratio of CD206+/CD11c+ macrophages (J) in the pituitary from mice treated with LPS (n = 3 mice). (K and L) qPCR analysis of the M2-like macrophage marker genes Mrc1 (K) and Cd301 (L) in the pituitary from mice treated with LPS. The injection dose of LPS was 0.5 mg/kg in (C–L). All data represent mean with SD. Statistical significance was determined by two-tailed Student’s t test, *p < 0.05, **p < 0.01, ***p < 0.001, ****p < 0.0001. The data underlying this figure can be found in S1 Data. IF, immunofluorescence. (TIF) [file pbio.3002403.s009.tif]
