# Supplementary material for: Single-cell transcriptomic analysis reveals rich pituitary–Immune interactions under systemic inflammation
Source: PLoS Biol. 2023 Dec 18;21(12):e3002403. doi: 10.1371/journal.pbio.3002403 (PMC10727439; doi:10.1371/journal.pbio.3002403)
Supplement: S1 Raw Images — (PDF) [file pbio.3002403.s010.pdf]

1. Original images (blots) of Fig 3F

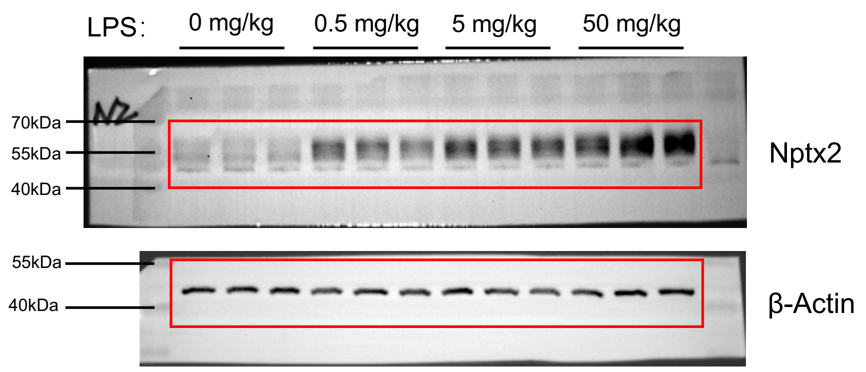

Imaging machine:
iBright™ CL1500 Imaging System
(Invitrogen)

2. Original images (blots) of S8I Fig

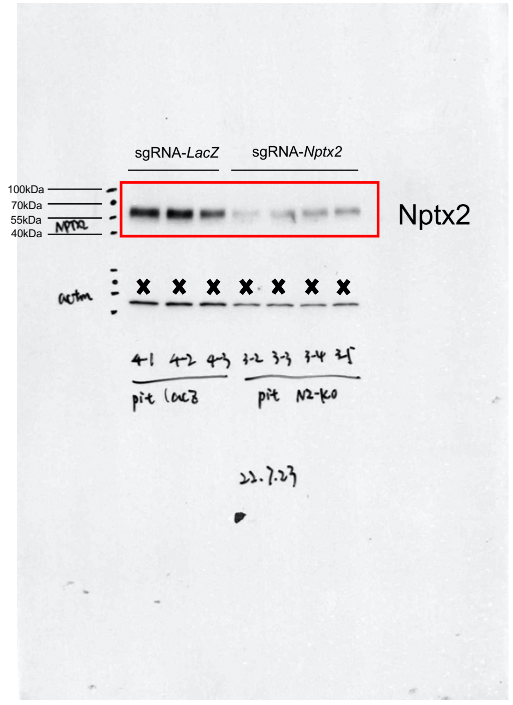

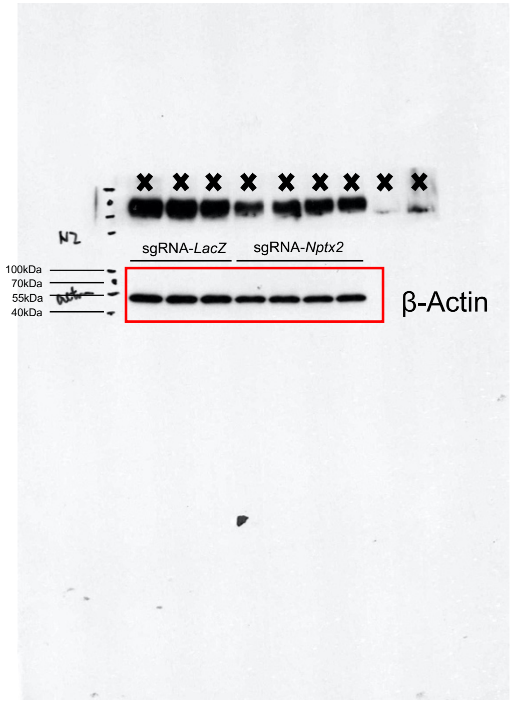

Imaging machine:
OPTIMAX 2010 X-Ray Film Processor
(PROTEC)
Exposure time: shorter time,
approximately 30 seconds.

Imaging machine:
OPTIMAX 2010 X-Ray Film Processor
(PROTEC)
Exposure time: longer time,
approximately 3 mins.
