# Supplementary material for: Single-cell transcriptomic analysis reveals rich pituitary–Immune interactions under systemic inflammation
Source: PLoS Biol. 2023 Dec 18;21(12):e3002403. doi: 10.1371/journal.pbio.3002403 (PMC10727439; doi:10.1371/journal.pbio.3002403)
Supplement: Attachment — Submitted filename: 2.PBIOLOGY-D-23-00680_R2_response to reviewer.pdf [file pbio.3002403.s025.pdf]

# Point-by-point Response

We appreciate the positive evaluations provided by the reviewers and their keen interest in our discoveries. Reviewer 1 pointed out that "This discovery is quite significant". Additionally, Reviewer 1 highlighted the remarkable aspect of our work "What's really impressive about this work is that the authors go on and provide mechanistic insights into how pituitary cells interact bidirectionally with immune cells to modulate protein secretion (either immune- or hormone-related)". Reviewer 2 acknowledged our study's analysis of the transcriptomic responses of pituitary HPCs, recognizing its potential as a resource for "the identification of biomarkers of sepsis-induced pituitary dysfunction". Reviewer 3 described our work as "novel and interesting". We greatly appreciate their assessments. At the same time, they raised several concerns and provided excellent suggestions. In the following section, we address each specific concern raised by the reviewers.

## Reviewer 1

*1.) The infiltration of neutrophils was demonstrated using flow cytometry which lacks spatial information and does not rule out the possibility of blood contamination or the accumulation of these cells at the brain borders. If these cells infiltrated into the parenchyma, were they only found in the pituitary? It is recommended to provide histology data to complement this finding.*

We thank Reviewer 1 for this suggestion. Our new experiments now reveal the spatial distribution of neutrophils in the revised manuscript (**S9F Fig**). The histology immunostaining of the experimental pituitary tissue was performed following thorough blood perfusion in mice, aiming to effectively eliminate circulating neutrophils. The proportion of neutrophils in peripheral blood mononuclear cells (PBMCs) of mice ranges from 10% to 30% [1, 2]. The absence of residual neutrophils in the blood vessels of the healthy mice indicates the success of our perfusion (**Figure R1, 0h**).

Analysis of the imaging data reveals that neutrophils migrate to the pituitary during inflammation, yet their presence is confined to the blood vessels in the pituitary and not detected within the pituitary parenchyma (**Figure R1A**). The observed adherence of neutrophils to the vessel wall indicates a process of neutrophil arrest mediated by chemokines [3]. The absence of neutrophils in the pituitary parenchyma could be a consequence of lower inflammation levels accompanied by minimal concentrations of chemokines, thus impeding neutrophil migration into the parenchyma.

Immunohistochemical staining of brain slices revealed that, under physiological conditions, neutrophils cannot be detected in the brain parenchyma, blood vessels, and meninges of perfused mice. However, under inflammatory conditions, a small number of neutrophils are also be detected at the meninges-like structures covering the brain (**Figure R1B, indicated by yellow arrows**). Additionally, a small number of neutrophils can be detected in the blood vessels within the brain parenchyma (**Figure R1B, indicated by white arrows**). Overall, the number of neutrophils in the pituitary is significantly higher than in the brain during the inflammatory state.

Combined with the Transwell assay (**Figure 4E**), our new data imply that hormone-producing cells (HPCs) in the pituitary exert a specific influence on neutrophil recruitment to the pituitary. To present our data more precisely, we have chosen to remove the term "parenchymal" from line 304 (in the original manuscript) of the main text to prevent potential confusion.

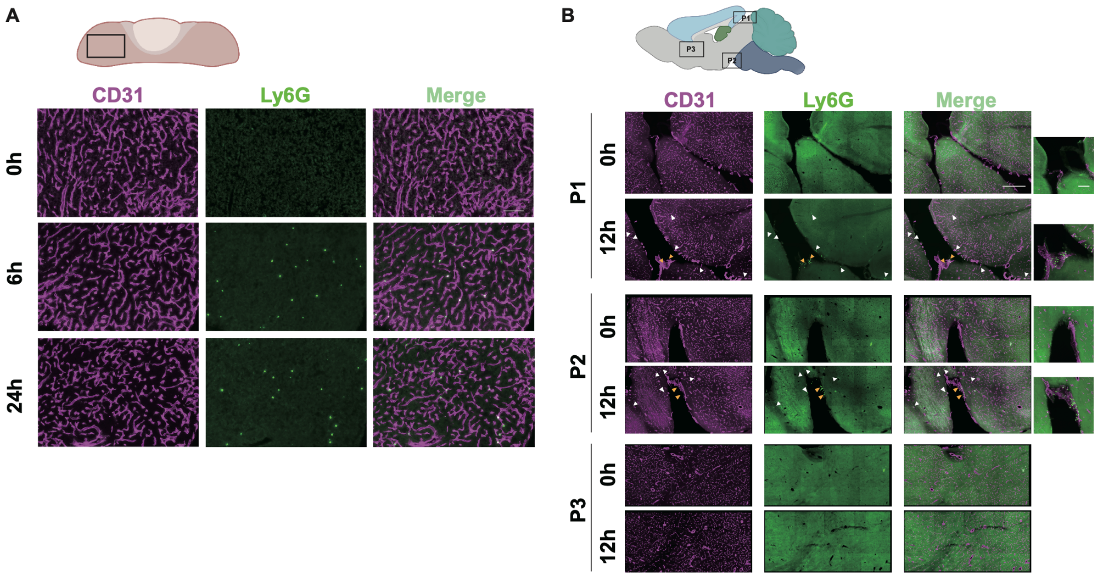


**Figure R1. Representative images showing CD31 (the marker of endothelial cells) and Ly6G (the marker of neutrophils) expression in the pituitary (A) and brain slices (B) from mice treated with saline and 0.5 mg/kg LPS at different time points.** White arrows indicate neutrophils located in the blood vessels within the parenchymal, and yellow arrows indicate neutrophils located in the meninges. The labels P1, P2, and P3 in (**B**) indicate different brain areas shown in the schematics. Scale bar, (A) 100 µm; (B) 400 µm; 100 µm (zoom in).


1. O'Connell KE, Mikkola AM, Stepanek AM, Vernet A, Hall CD, Sun CC, et al. Practical murine hematopathology: a comparative review and implications for research. Comp Med. 2015;65(2):96-113. PubMed PMID: 25926395; PubMed Central PMCID: PMCPMC4408895.
2. Eruslanov EB, Singhal S, Albelda SM. Mouse versus Human Neutrophils in Cancer: A Major Knowledge Gap. Trends Cancer. 2017;3(2):149-60. Epub 20170119. doi: 10.1016/j.trecan.2016.12.006. PubMed PMID: 28718445; PubMed Central PMCID: PMCPMC5518602.
3. Ley K, Laudanna C, Cybulsky MI, Nourshargh S. Getting to the site of inflammation: the leukocyte adhesion cascade updated. Nat Rev Immunol. 2007;7(9):678-89. doi: 10.1038/nri2156. PubMed PMID: 17717539.


*2.) The section on Crhr1 is a bit confusing as some of the provided data suggest that LPS causes an increase in hormone secretion, as evidenced by the enrichment of the "positive regulation of secretion" term in DEGs, with Crhr1 being one of the representative genes (line 180). However, the downregulation of Crhr1(Fig S5H) in the LPS-treated group seems to contradict this finding. The authors should clarify the interpretation of this data. Additionally, it would be helpful to provide clearer information about the directionality of regulation on Lines 185-186.*

We thank Reviewer 1 for bringing up the need for clarification. Initially, our aim was to identify the specific and the most perturbed pathway in corticotropes. Therefore, we performed GO pathway enrichment analysis using all differentially expressed genes (DEGs) specific to corticotropes, including both upregulated and downregulated genes. We agree that the inclusion of both sets of DEGs for GO term analysis would produce misleading implications of regulation directionality.

To enhance clarity, we performed a separate GO enrichment analysis using the corticotrope-specific upregulated DEGs and downregulated DEGs. As depicted in **Figure R2A** and **R2B**, the highly enriched pathways among the upregulated DEGs primarily involve protein modification (**Figure R2A** and **R2C**), while the highly enriched pathways among the downregulated DEGs primarily involve nucleoside triphosphate metabolic processes (**Figure R2B**). Though the signaling pathways of "negative regulation of kinase activity" and "positive regulation of secretion" do not appear at the forefront of the GO list, it’s important to note that they are still significantly enriched according to the GSEA analysis (**Figure R2D** and **R2E**).

Furthermore, in the revised manuscript, we have presented the newly analyzed corticotrope-specific upregulated and downregulated signaling pathways as shown in **Figure R2A and R2B**, along with representative genes (**S6 Fig**). Additionally, we have removed original data from **S5 Fig** (in the original manuscript) and integrated the *Crhr1*-related results into **S8 Fig** in the revised manuscript.

Regarding the specific concern on *Crhr1* expression, we believe its downregulation reflects the effects of negative feedback. It has been well-established that CRHR1 activates the cAMP-ERK1/2 pathway upon CRH stimulation, which in turn promotes the expression of *Pomc* gene and thus the expression of its encoded precursor protein for ACTH [4]. Previous reports have indicated that corticosterone administration can inhibit *Crhr1* expression as part of a negative feedback mechanism [5]. In the inflammation model, ACTH significantly upregulates corticosterone levels. Additionally, *Crhr1* exhibits specific expression in corticotropes compared to other HPCs in the pituitary (**S8C-S8D Fig** in the revised manuscript). Thus, the downregulation of *Crhr1* serves as an illustrative example highlighting the specificity of corticotropes during inflammation.

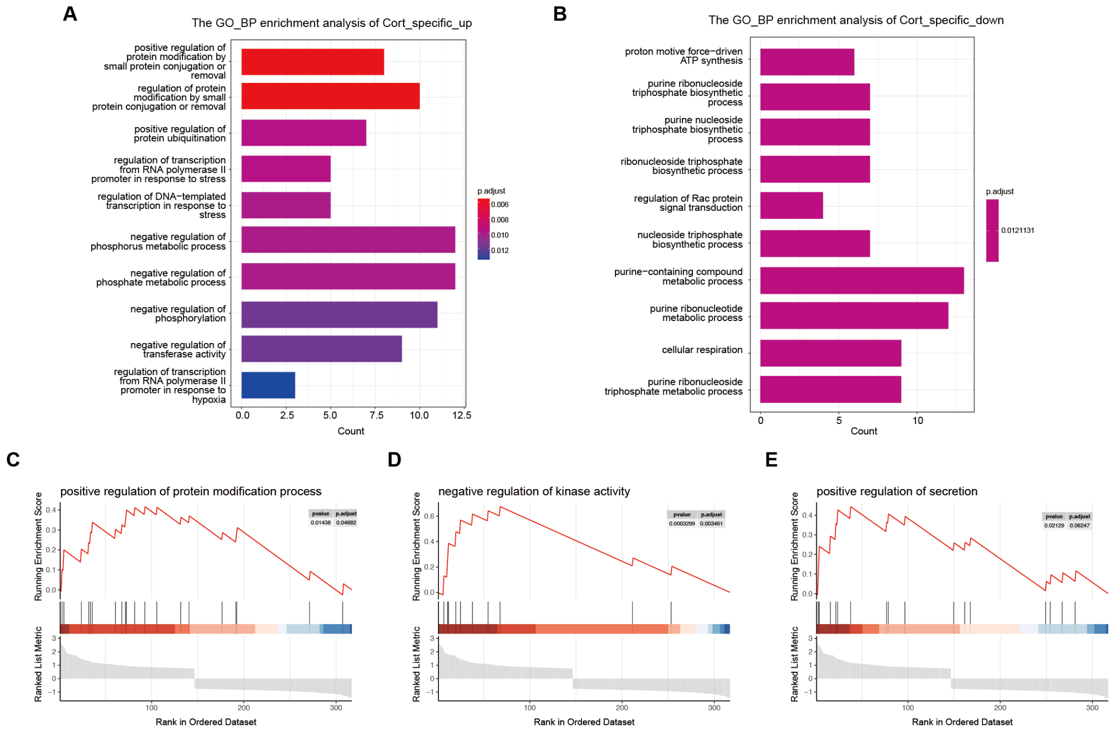


**Figure R2. (A** and **B) GO analysis on unique DEGs of corticotropes during inflammation revealing the upregulated pathways (A) and the downregulated pathways (B). (C-E) GSEA profiles showing significant enrichment of gene sets in upregulated GO pathways.**

4. Jenks BG. Regulation of proopiomelanocortin gene expression: an overview of the signaling cascades, transcription factors, and responsive elements involved. Ann N Y Acad Sci. 2009;1163:17-30. doi: 10.1111/j.1749-6632.2008.03620.x. PubMed PMID: 19456325.

5. Uriguen L, Arteta D, Diez-Alarcia R, Ferrer-Alcon M, Diaz A, Pazos A, et al. Gene expression patterns in brain cortex of three different animal models of depression. Genes Brain Behav. 2008;7(6):649-58. Epub 20080321. doi: 10.1111/j.1601-183X.2008.00402. x. PubMed PMID: 18363858.

*3.) On Line 200, the authors mentioned proteomic analysis but did not provide a description of how it was performed. It would be helpful to include more information about the methods used for this analysis. Additionally, it is not clear whether the cytokines/chemokines that show upregulation in the transcriptomic data are also detected in the proteomic dataset. Clarification on this point would be valuable.*

We thank Reviewer 1 for asking us to clarify the methods of proteomics. We have now added detailed information, as we describe below. "**Protein sample preparation for LC-MS/MS analysis**. Mice were i.p. injected with saline or 50 mg/kg LPS, and the pituitary was collected at 6 hours after LPS treatment. Ice-cold lysis buffer (500 μL 8M Urea with 1x protease inhibitors cocktail) was used to extract protein from the pituitary, and the tissue was manually homogenized with Dounce grinder and ultrasonication, then lysates were centrifuged at 16,000g for 10 min at 4°C. The supernatant was collected, and the total protein was determined by the BCA assay kit (Solarbio, PC0020). Protein was reduced in 5 mM dithiothreitol (DTT, Sigma-Aldrich) at 37 °C for 1 hour, followed by alkylation with 20 mM iodoacetamide (Sigma-

Aldrich) in dark for 1 hour, then added DTT again for another 15 min. Added 8 M urea to the sample up to 1 mL. After centrifugation, transferred the supernatant to a 10 KDa molecular weight cut-off (MWCO) filter tube (Millipore) and centrifuged for 10 min, then the concentrated protein in upper reservoir was washed once with 8M urea and once with 50 mM $NH_4HCO_3$. Resuspended the sample with 200 μL 100mM $NH_4HCO_3$ and added 100 μL 100 ug/mL sequencing-grade trypsin (Promega), then digested at 37 °C for 12 hours. After digestion, centrifuged at 14,000g for 10 min and collected the solution and dried in a vacuum centrifuge. Samples were resuspended in 100 μL of 0.1% formic acid (FA) before desalted with C18 stationary phase (Omix, Agilent). The samples dried down and resuspended in 5 μL of 0.01% FA before LC-MS/MS analysis. **LC-MS/MS analysis**: The LC-MS/MS experiments were performed on Fusion03 (Thermo Scientific Orbitrap Fusion Lumos Tribrid Mass Spectrometer). The MS raw data were analyzed by PEAKS Studio v8.5 protein database containing 4136 entries. Trypsin was set as an enzyme. The protein expression data were filtered to have at least 70% valid abundance values in the same treatment group. Log2(Fold changes) >1 or <-1."

Reviewer 1 asked us to compare between proteomics and transcriptomics. We have listed several genes encoding secreted proteins, including *Serpina3n*, *Isg15*, and *Lgals9*, which can also be identified in the proteomic dataset (**S12 Table**). These genes exhibit a high basal expression level in the transcriptomic dataset and can be further upregulated under inflammatory conditions. It is important to note that not all genes encoding secreted proteins can be detected in the proteomic dataset. One possible reason for this discrepancy is that proteomic techniques often have lower detection sensitivity and a limited dynamic range, which can lead to failures in detecting changes in low-expressed proteins or variations in protein abundance. Furthermore, in the handling of protein samples, we utilized centrifugal columns with a 10 kDa molecular weight cutoff, this approach might have hindered the detection of proteins smaller than 10 kDa, including CXCL1 and other chemokines.

*4.) The terminology for M1/M2 macrophage is considered to be obsolete, and it may be more appropriate to use marker genes such as Mrc1+ to describe the population. This would be more reflective of the current understanding of macrophage polarization.*

We agree. We have revised our terminology accordingly. In the revised manuscript, we substituted the terminologies M1 or M2 with a specific cell surface marker. For our Fluorescence-Activated Cell Sorting (FACS) study, we utilized $CD206^{+}CD11c^{-}$ to designate M2-like macrophages, while $CD206^{-}CD11c^{+}$ was employed to characterize M1-like macrophages. At the transcriptional level, we utilized $Mrc1^{+}$ and $CD301^{+}$ to delineate M2-like macrophages.

## Reviewer 2

*The study design is overcomplicated, some experiments confusing or unnecessary, and the manuscript seems to be in some parts defocused. Because of that, the manuscript reads as 2-3 separate papers, with different degrees of finalization.*

We would like to thank Reviewer 2 for pointing out the perceived complexity of the study design. We understand that the manuscript may come across as multifaceted, but our intention was to comprehensively explore the pituitary's transcriptomic response under inflammation. We structured our investigation around three interrelated questions: 1) The responses of pituitary HPCs to inflammatory stimuli; 2) The potential secretion of additional factors by pituitary HPCs beyond traditional hormones; 3) The broad connections between the pituitary and immune system.

Our approach integrates single-cell transcriptomic analysis with selected experimental validations to provide a holistic view of the pituitary's role in regulating inflammatory responses. We hope that the dense dataset helps convey essential insights into understanding the pituitary response to inflammation. Guided by reviewers' advice, we have revised the manuscript to enhance its focus and clarity to ensure that the interconnected nature of the questions is evident to all readers.

*1.) The study starts with a bulk RNAseq analysis of the pituitary transcriptome changes during LPS- and CLP-induced-mediated inflammation. Analysis of those experiments provided rather expected results, which are not organ-specific such as apoptosis, and generally pointing to tissue inflammation (increased cytokines and their signaling pathways). Unfortunately, except for the "myeloid leukocyte migration pathway - chemokines", other pathways identified in these experiments were not studied further in a cell-specific manner later.*

We agree that many of signaling pathways associated with the pituitary transcriptome changes do not exhibit clear tissue specificity, and show enrichments in inflammatory responses. However, such findings are important in two aspects. First, there have been no systematic studies on the transcriptomic effect of LPS-induced inflammation on pituitary HPCs. Our observations of cell type-specific responses highlight the importance of characterizing such responses. Second, pituitary HPCs specialize in secreting peptide hormones that regulate physiological functions throughout the body. Since cytokines, which include chemokines, are protein factors like traditional pituitary hormones, it's crucial to investigate if HPCs can release these cytokines and other factors to modulate body physiology during inflammation. Our findings of cytokines and Nptx2 highlight the value of such approach. In the analysis of Gene Ontology (GO) and Kyoto Encyclopedia of Genes and Genomes (KEGG), the top-ranked pathways are primarily associated with "cytokine generation", indicating the crucial role of the pituitary's secretory function and its interaction with immune cells during inflammation. Therefore, we have provided descriptive text for representative pathways in the manuscript, highlighting the "secretion" pathway and conducting an in-depth exploration of the "myeloid leukocyte migration pathway - chemokines."

*2.) In addition, no clear benefit to the main story of the manuscript came from the comparison of LPS with CLP experiments (meaning this part could also be removed). Unless providing additional data would be considered. For example, it would be of interest to find similarities and differences between the top DEG (differentially expressed genes) in pituitaries of LPS- and CLP-treated mice.*

We agree that the LPS model is more productive than the CLP model in this study. To explore the pituitary's response to immune challenges, we first selected two widely used models to mimic systemic inflammation: LPS and CLP. After CLP, intestinal microbiota enters the abdominal cavity, triggering systemic inflammation with polymicrobial infection, which produces immune, hemodynamic, and biochemical responses similar to human sepsis [6, 7]. Therefore, CLP can simulate systemic inflammation more physiologically. Our sequencing results showed that more than three-quarters of the upregulated genes in the CLP group could also be found in the LPS group, and more than two-thirds of the downregulated genes were shared between the two groups (**Figure R3**). The similarities between the LPS and CLP groups were high in GO and KEGG analysis (**Figure R4 and R5**). The findings indicate a striking similarity in the pituitary response to endotoxin and bacterial-induced inflammatory reactions. We have included the TPM data from RNA sequencing experiments related to LPS and CLP treatments in **S1 Table** within the original manuscript. We believe that these resources can provide additional information about the response of the pituitary to sepsis. Following Reviewer 2's advice, we have revised the manuscript to highlight the similarities between CLP and LPS treatments (see also our responses to the following concerns).

However, it has been challenging to control the amount of intestinal microbiota leakage in the CLP model, which results in a wide range of variation in modeling outcomes. As a result, the CLP model is less easily standardized than the LPS model. Therefore, we focused on LPS treatment to simulate systemic inflammatory responses caused by bacterial infections, specifically for investigating pituitary function.

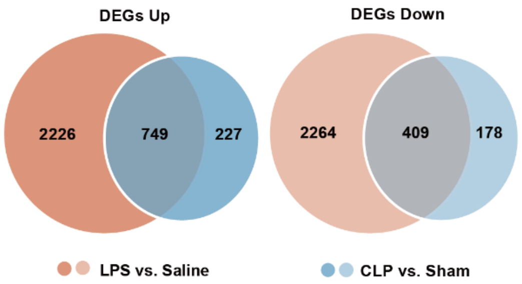


**Figure R3. Venn diagrams showing the intersection of DEGs in the pituitary under LPS and CLP treatments.** Left panel: the intersection of upregulated genes; Right panel: the intersection of downregulated genes.

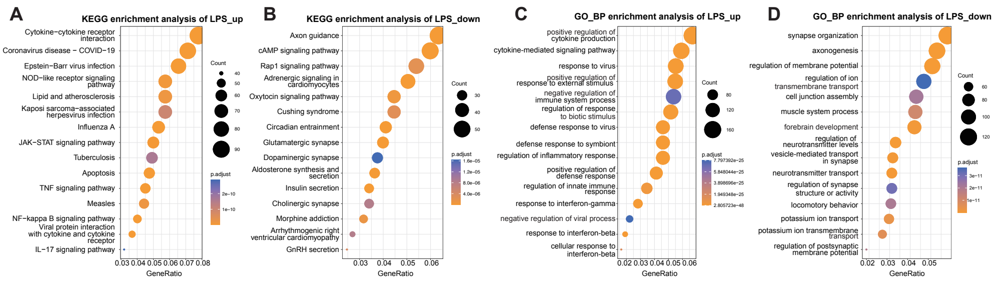


**Figure R4. KEGG (A and B) and GO (C and D) analyses of pituitary DEGs after saline and LPS treatment.**

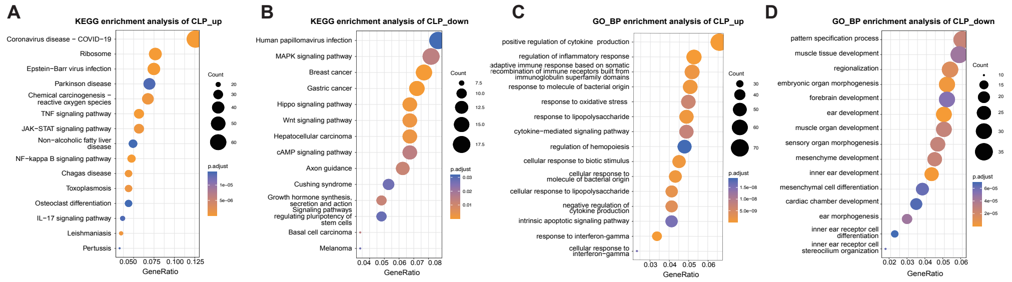


**Figure R5. KEGG (A and B) and GO (C and D) analyses of pituitary DEGs after Sham and CLP treatment.**

6. Wichterman KA, Baue AE, Chaudry IH. Sepsis and septic shock--a review of laboratory models and a proposal. J Surg Res. 1980;29(2):189-201. doi: 10.1016/0022-4804(80)90037-2. PubMed PMID: 6997619.

7. Rittirsch D, Huber-Lang MS, Flierl MA, Ward PA. Immunodesign of experimental sepsis by cecal ligation and puncture. Nat Protoc. 2009;4(1):31-6. doi: 10.1038/nprot.2008.214. PubMed PMID: 19131954; PubMed Central PMCID: PMCPMC2754226.

*3.) In the end, LPS-driven systemic inflammation is TLR-4 dependent whereas CLP-induced peritonitis, a more physiological mouse model of sepsis, is driven by whole bacteria activating multiple TLRs and other PRRs. Afterward, those results could be compared to scRNA seq results from different subsets of pituitary HPCs.*

We performed correlation analyses between the transcriptomes of six HPC types obtained from the single-cell analysis after LPS treatment and the bulk transcriptome data. The correlation fitting curve demonstrates a clear positive correlation between the transcriptomes of individual HPCs in the pituitary and the bulk transcriptome data under both LPS and CLP treatments (**Figure R6**). The correlation analysis data indicates that HPCs in the pituitary play crucial roles in the response to inflammation. In the revised version, we have included the correlation curve depicting the relationship between the single-cell analysis and bulk transcriptome data of LPS (**S2F Fig**).

As mentioned above, while CLP serves as a more physiological model of human systemic inflammation, both LPS and CLP can effectively induce robust systemic inflammation in the organism, exhibiting comparable effects on the pituitary at the transcriptional level. Nevertheless, in comparison to the CLP model, LPS treatment offers greater convenience in administration and allows for better control of the inflammatory process.

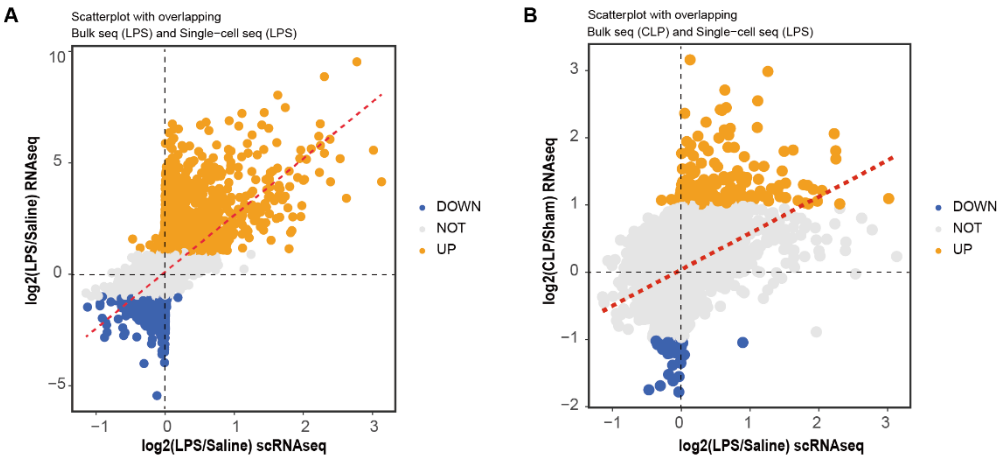


**Figure R6. Scatterplots showing the correlation between scRNA-seq from LPS treatments and bulk RNA-seq from LPS treatment (A) and CLP treatment (B).**

*4.) There are also only four pathways presented in both conditions. Are they no other significantly enriched gene sets (pathways) in both groups (CLP & LPS)? It has been shown that elevated GCs concentrations can negatively influence the pituitary function through e.g. negative feedback induction, suppression of PC1/3, which enzyme process POMC into ACTH, and via suppressing ACTH release through upregulation of Annexin A1. Therefore, it would be of interest to see whether there are these or other GC-regulated genes (DEG) present in those datasets (e.g. Gliz, etc).*

**Figures R4** and **R5** illustrate additional signaling pathways enriched in the pituitary after inflammation, such as “response to interferon-gamma”. Due to space limitations, we have selected only a few representative pathways for display. In the revised manuscript, we have described the additional pathways following inflammatory challenges.

Following Reviewer 2’s suggestion, we examined the “Response to GC” pathway and find it is also upregulated (**Figure R7**). Here, we present the GSEA data for the “Response to GC” pathway after LPS and CLP treatments. After LPS treatment, the expression level of *Gilz* (glucocorticoid-induced leucine zipper) was upregulated, whereas no significant change was observed after CLP treatment (**Figure R8**).

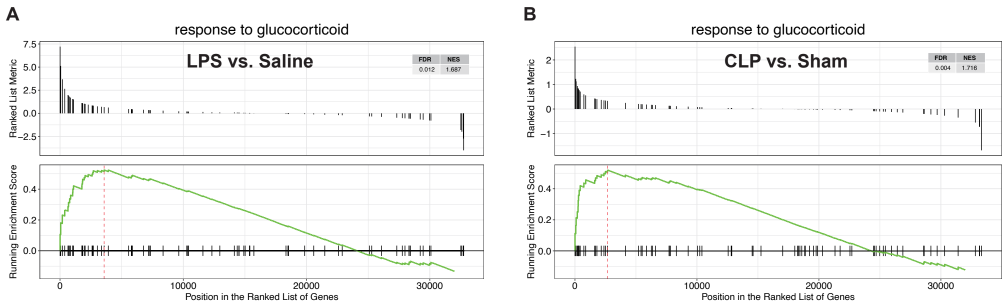


**Figure R7. GSEA profiles showing enrichment of "response to glucocorticoid" gene set after LPS (A) or CLP (B) treatment in the pituitary.**

| gene | group | log2(FC) | P-value | P-adj |
|---|---|---|---|---|
| Gilz | LPS vs. Saline | 0.698961 | 6.19E-06 | 6.44E-05 |
| Gilz | CLP vs. Sham | 0.199894 | 0.360171 | 0.669309 |

**Figure R8. The gene expression of *Gilz* after LPS or CLP treatment.**

*5.) What was the rationale for using the 6h time point? Usually, for gene expression, 2-3 hours are sufficient and mark the top of transcriptomic responses. Initial bell-like response (max expression) after LPS injection is rather expected, however, it usually occurs at 2-3-hour time points. Therefore, I wonder why this time was not included in the analysis.*

We selected this time point based on the Seemann et al. (2017) study, which demonstrated the peak of Clinical Severity Score (CSS) in mice after LPS or CLP treatment at 6 hours [8]. To ensure the physiological relevance, stability, and reproducibility of our experimental results, we conducted the transcriptomic analysis of the pituitary gland 6 hours after inducing inflammation. In the revised manuscript, we have cited the study by Seemann et al. and briefly explained our rationale for choosing the 6-hour time point.

8. Seemann S, Zohles F, Lupp A. Comprehensive comparison of three different animal models for systemic inflammation. J Biomed Sci. 2017;24(1):60. Epub 2017/08/25. doi: 10.1186/s12929-017-0370-8. PubMed PMID: 28836970; PubMed Central PMCID: PMCPMC5569462.

*6.) Besides p-values, GSEA pathways should also contain information about NES (nominated enrichment scores) and FDR (false detection rates).*

Thanks. We have provided the information (**Figure R9** below) in our revised manuscript (**Figure 1B** and **S1C Fig**).

A. LPS goGSEA

| ID | Description | setSize | enrichmentScore | NES | pvalue | p.adjust | qvalue |
|---|---|---|---|---|---|---|---|
| GO:0097529 | myeloid leukocyte migration | 228 | 0.66459811 | 2.44658724 | 0.00012143 | 0.00199295 | 0.00139592 |
| GO:2001235 | positive regulation of apoptotic signaling pathway | 155 | 0.54707375 | 1.93177946 | 0.00012893 | 0.00199295 | 0.00139592 |
| GO:0001819 | positive regulation of cytokine production | 483 | 0.70091528 | 2.73144486 | 0.00010997 | 0.00199295 | 0.00139592 |
| GO:0050729 | positive regulation of inflammatory response | 140 | 0.69317144 | 2.41368217 | 0.00013173 | 0.00199295 | 0.00139592 |
| GO:1901216 | positive regulation of neuron death | 131 | 0.51131653 | 1.76721197 | 0.00013342 | 0.00199295 | 0.00139592 |

B. CLP goGSEA

| ID | Description | setSize | enrichmentScore | NES | pvalue | p.adjust | qvalue |
|---|---|---|---|---|---|---|---|
| GO:0097529 | myeloid leukocyte migration | 226 | 0.55999451 | 2.12483659 | 0.00013459 | 0.00263583 | 0.00191068 |
| GO:2001235 | positive regulation of apoptotic signaling pathway | 153 | 0.48468144 | 1.76077518 | 0.00014294 | 0.00263583 | 0.00191068 |
| GO:0001819 | positive regulation of cytokine production | 479 | 0.57188302 | 2.30980593 | 0.00012083 | 0.00263583 | 0.00191068 |
| GO:0050729 | positive regulation of inflammatory response | 140 | 0.62720318 | 2.25574377 | 0.0001442 | 0.00263583 | 0.00191068 |

**Figure R9. The detailed information of GSEA profiles.**

*7.) Please provide also more details about your CLP procedure in the Materials and Methods section.*

We have included additional details about the CLP procedure in the **Materials and Methods** section of our revised manuscript. Following is the detailed method for the CLP procedure: “The mice were deeply anesthetized with 250 mg/kg of tribromoethanol via i.p. injection. The abdominal fur was carefully removed using a razor blade to expose the abdominal skin. The mice were positioned on a clean surgical operating table, and a clean surgical blade was used to make a midline incision in the abdominal skin, creating a 1-2 cm opening. The peritoneum was meticulously dissected to expose the cecum and the connected intestine. Using a medical suture, a ligature was applied approximately halfway up from the bottom of the cecum. Next, the cecum was punctured using a 21G needle, creating 2-3 punctures, and gently squeezed to expel a small amount of feces. Subsequently, the cecum and the adjacent intestine were placed back into the mouse's abdominal cavity, and the peritoneum was sutured using a medical suture. Used 7 mm mouse wound clips to secure the abdominal skin. For the control group mice, steps 4 and 5 were omitted.”

*8.) In order to analyze an individual response of the pituitary HPCs to inflammation, a scRNA seq was performed on pituitary cells isolated from mice undergoing either LPS- (conc. 0.5-50 mg/kg & time 3-504 hrs. "3wks"), poly I:C- or TNF-alpha- induced systemic inflammation. Results of this study demonstrated a clear separation of different HPCs within pituitary gland microenvironment and indicated "healthy" and "inflamed" status of different pituitary HPCs subsets. Moreover, it indicated that corticotropes and somatotrophs showed the highest transcriptomic responses during systemic inflammation. Based on which DEG sets were those two cellular states defined? proinflammatory cytokines, chemokines or other factors? Were the DEGs sets used to characterize "inflammatory state" the same among different pituitary HPCs subsets? Meaning the same cytokines/chemokines etc. This needs to be better clarified and depicted in the figure.*

We thank Reviewer 2 for raising this important question. It is essential to clarify that the cellular states ("inflammatory" or "healthy") were defined using unsupervised clustering analysis with the Louvain algorithm. The identification of DEGs was conducted only after these clusters had been defined. Therefore, the characterization of “inflammatory state” did not involve *a priori* selection of DEGs.

As depicted in the upset plot in **Figure 2D**, the DEGs identified between the "inflammatory" and "healthy" states varied across different HPC subsets. This observation is not unexpected, considering that HPCs have distinct transcriptional lineages, leading to potentially different gene regulatory patterns under systemic inflammatory challenges.

*9.) Considering differences in the inflammatory responses induced by LPS, poly I:C- and TNF-alpha, it would be of great interest to analyze and compare the number of DEGs and their interactions (GSEA, etc.) individually and between each other in each pituitary HPCs subsets.*

We thank Reviewer 2 for the valuable suggestion. While it would indeed be of great interest to analyze and compare the number of DEGs and their interactions (GSEA, etc.) individually and between each pituitary HPC subset for LPS, Poly (I:C), and TNF-α groups, we encountered challenges due to differences in cell numbers among these groups. The varying cell numbers in each HPC population between the treatment groups could influence the number of DEGs identified, potentially leading to less meaningful insights. Similarly, downstream analyses, such as GSEA, might be affected by the differences in cell numbers and might not produce robust and interpretable outcomes.

We appreciate the reviewer's understanding of these limitations. Despite these challenges, we have carefully addressed the differences in responses induced by each treatment and presented our findings accordingly.

*10). The analysis of how different concentrations of P IC influence the number of healthy vs. inflamed cells does not bring much information (Fig.S3).*

The analyses presented in **S3 Fig** were originally intended to classify the HPCs from Poly (I:C) or TNF-α treatment groups into binary cell states. However, we understand that the information provided may not have been as informative as intended. In response to this valuable feedback, we have incorporated an alluvial plot to better demonstrate the classification of HPCs into healthy and inflamed cell states under different concentrations of Poly (I:C) treatment (**S3E Fig** in the revised manuscript; **Figure R10** below). We agree that this improvement in visualization provides a clearer and more comprehensive representation of the effects of varying concentrations of Poly (I:C) on the cellular states.

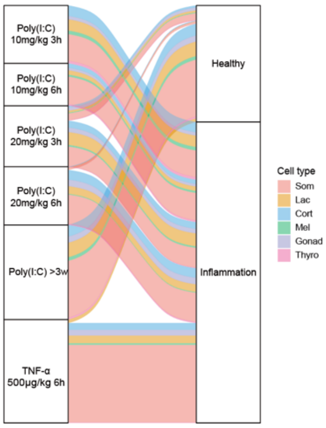


**Figure R10. Alluvial plot showing the cell state distribution of six HPCs under poly (I:C) and TNF-α treatment groups.**

*11.) Figure 3 of the manuscripts shows a number of conserved and differentially expressed "marker" genes from each pituitary HPC set. The expression of the latter genes was then compared between different organs by qPCR. I am not sure what identification of the conservative genes in each pituitary HPC subset brings to the main story. Moreover, their conservative status is not clear to me. For example, the Edil3 gene has been identified as a "conservative" gene for lactotrophs. But Del-1 encoded by this gene is the negative regulator of leukocyte adhesion to endothelial cells, through blocking interactions of beta 2 integrin with ICAMs. It is expressed in restricted organs and cell types including the brain, eye, or adrenals, where its expression is strongly suppressed during LPS-induced inflammation. In other words, it is hard to imagine that in the lactotrophs, its expression would not be altered by inflammation.*

We appreciate Reviewer 2's valuable feedback and insights into specific marker genes like *Edil3*, which enrich our understanding of the complex molecular dynamics within pituitary cell subsets. The identification of conserved marker genes in each pituitary HPC subset serves a critical purpose in accurately characterizing cell types and studying their responses under systemic inflammation. The presence of conserved markers that align with previously identified HPC markers adds confidence to the cell identities annotated in our study (**Figure 2**).

Regarding the specific example of the *Edil3* gene, we thank Reviewer 2 for providing additional insights into its functions and expression patterns. Indeed, *Edil3* has been previously identified as a marker gene for lactotropes [9], which aligns with its abundant expression in lactotropes within our dataset. While *Edil3*'s functions as an inflammation regulator have been mainly studied in endothelial cells [10], its roles and expression in pituitary lactotropes remain less explored and may not follow the same patterns as in endothelial cells. In this context, it is essential to recognize that our study focuses on the transcriptomic responses of pituitary HPCs under systemic inflammation, and specific functions of marker genes like *Edil3* within pituitary cell types are beyond the scope of this investigation. Furthermore, our bulk RNA-seq results indicate that the *Edil3* gene in the pituitary did not exhibit significant changes after LPS treatment (**Figure R11**). This observation supports our findings and suggests that *Edil3*'s expression in the pituitary may not be strongly affected by inflammation.

| gene | group | log2(FC) | P-value | P-adj |
|---|---|---|---|---|
| Edil3 | LPS vs. Saline | -0.02291 | 0.870894 | 0.942309 |

**Figure R11. The gene expression of *Edil3* after LPS treatment.**

9. Cheung LYM, George AS, McGee SR, Daly AZ, Brinkmeier ML, Ellsworth BS, et al. Single-Cell RNA Sequencing Reveals Novel Markers of Male Pituitary Stem Cells and Hormone-Producing Cell Types. Endocrinology. 2018;159(12):3910-24. Epub 2018/10/20. doi: 10.1210/en.2018-00750. PubMed PMID: 30335147; PubMed Central PMCID: PMCPMC6240904.
10. Li M, Zhong D, Li G. Regulatory role of local tissue signal Del-1 in cancer and inflammation: a review. Cell Mol Biol Lett. 2021;26(1):31. Epub 20210703. doi: 10.1186/s11658-021-00274-9. PubMed PMID: 34217213; PubMed Central PMCID: PMCPMC8254313.

*12.) Next part of the manuscript (2nd story), focuses on the identification of Nptx2 as an inflammatory marker gene of corticotropes. Previously, decreased levels of Nptx2 have been identified as a prognostic biomarker of Alzheimer's disease and schizophrenia (or in general as accelerated cognitive decline). Meanwhile, overexpression of Nptx2 was sufficient to restrain complement activity (C1q) and ameliorate microglia-mediated synapse loss. Those results indeed demonstrate that Nptx2 plays an important role in the "health" status of neuronal cells. Interestingly, qPCR data from Fig.3D showing decreased expression of this gene in the cortex and midbrain seems to be in favor of the above data. However, the question remains whether actually, pituitary gland is the only "sole" contributor of Nptx2 in the plasma during inflammation. Often mRNA changes at a given time may not necessarily reflect the actual serum protein levels. For example, expression of the Nptx2 gene might have been induced at the earliest time point in certain organs.*

We appreciate Reviewer 2's valuable comments on the identification of Nptx2 as an inflammatory marker gene of corticotropes. We agree that mRNA changes at a given time may not fully reflect actual serum protein levels, and it is crucial to determine whether the pituitary gland is the major contributor to Nptx2 in the plasma during inflammation. We have conducted several analyses to shed light on this matter, and two lines of evidence strongly support the pituitary as the major source of Nptx2 in the plasma during inflammation.

Firstly, our qPCR results (**Figure 3D**) revealed very low baseline *Nptx2* mRNA expression levels in peripheral organs under physiological conditions. These findings align with data from The Human Protein Atlas database (**Figure R12**). Additionally, after LPS treatments, *Nptx2* expression levels significantly increased in the pituitary, remained relatively low in other major peripheral organs, and were significantly downregulated in the brain. These observations suggest that the pituitary likely represents the primary source of Nptx2 in the plasma in response to inflammation.

Secondly, we employed the AAV-mediated CRISPR/Cas9 genome editing system to disrupt Nptx2 expression specifically in the pituitary. Stereotaxic microinjection of AAV vectors into the mouse pituitary significantly reduced Nptx2 protein expression in the pituitary and led to a notable decrease in serum Nptx2 protein levels (**S8I** and **S8J Fig**). These results highlight the crucial contribution of the pituitary to serum Nptx2 during inflammation.

Thus, by combining measurements of *Nptx2* mRNA levels using qPCR and protein levels using ELISA, along with pituitary-specific *Nptx2* knockout *in vivo*, we can confidently conclude that the pituitary gland is the primary contributor to serum Nptx2 during LPS treatment.

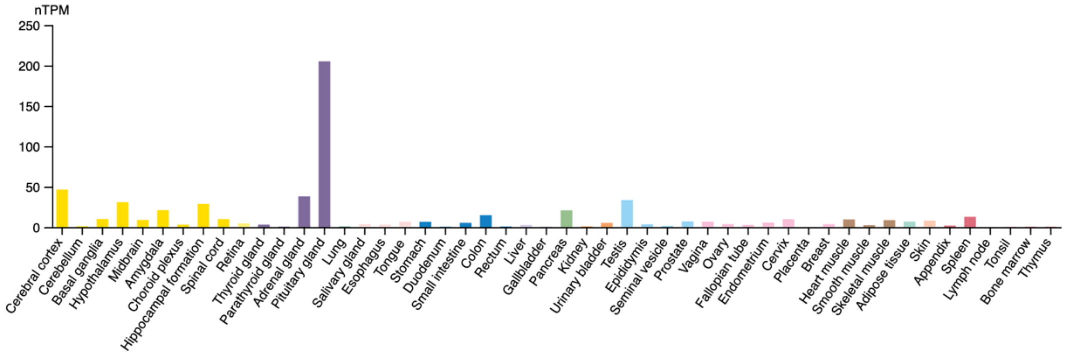


**Figure R12. The gene expression of *Nptx2* in various organs from The Human Protein Atlas database.** (https://www.proteinatlas.org/ENSG00000106236-Nptx2 /summary/rna)

*13.) Were all data (including qPCR) shown in Fig. 3 driven from 6 hours time point after LPS injection? If yes, it could have been stated as 1 sentence at the end of Fig.3's legend.*

Thanks. We have revised accordingly to clarify the concern.

*14.) Moreover, looking at Fig. 3E, it seems that although the Nptx2 gene expression was found in Pomc expressing cells, and it increased a bit after LPS injection (0.5 mg/kg), it did not markedly change when higher concentration (inflammation) was tested (40 mg/kg). It doesn't correspond to WB results showing a strong upregulation of Nptx2 in the pituitary after 50 mg/kg of LPS. Of interest, there is no information about 40 mg/kg of LPS treatment in the legends of Figure Was it 40 or 50 mg/kg?*

We appreciate Reviewer 2 for raising the need for clarification. We have provided clearer, zoomed-in images of the relevant data along with statistical information (**Figure R13** below; **Figure 3E and S8H Fig** in the manuscript). These new figures present a more accurate representation of *Nptx2* gene expression in POMC-expressing cells after LPS injection at different concentrations. Furthermore, we acknowledge the oversight in the figure legend, and we have now supplemented the information to include details about the 40 mg/kg LPS treatment.

Upon closer examination of the new data, it is evident that *Nptx2* gene expression indeed increases in POMC-expressing cells in a dose-dependent manner after LPS injection. These findings align with the Western blot results showing a robust upregulation of Nptx2 in the pituitary after 50 mg/kg of LPS. With these updates, we believe that the data presented in our revised manuscript more accurately reflect the effects of different LPS concentrations on *Nptx2* gene expression in POMC-expressing cells.

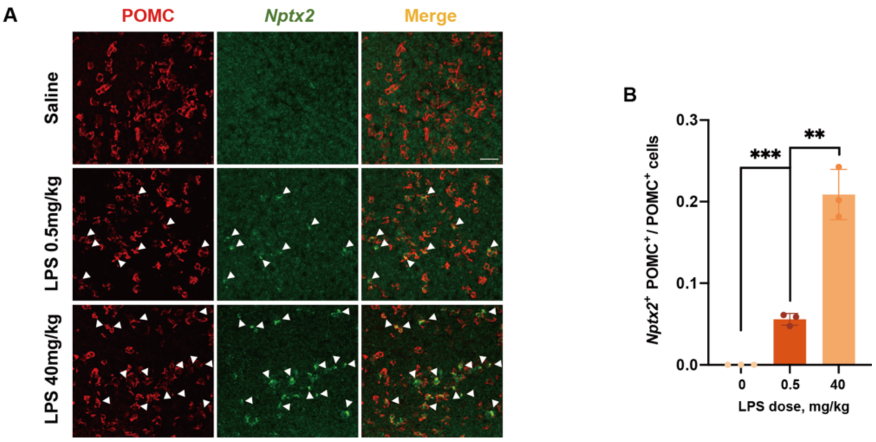


**Figure R13. (A) Representative images showing ISH of *Nptx2* RNA (green) and IF of POMC (red) in the pituitary from mice treated with saline, 0.5 mg/kg or 40 mg/kg LPS for 6 hours.** Arrows indicate cells co-expressing *Nptx2* and POMC. Scale bar, 50 µm. **(B) Histogram showing the statistic information of the percentage of *Nptx2*$^{+}$POMC$^{+}$ cells in POMC$^{+}$ cells.** Statistical significance was determined by two-tailed Student's t-test, **$p<0.01$, ***$p<0.001$.

*15.) As presented in Fig. S7G serum Nptx2 decreased partly after silencing of the Nptx2 gene in the pituitary suggesting that indeed pituitary gland might contribute to its systemic levels, however, it was not fully alleviated. Thus, Nptx2 measurement after LPS injections into hypophysectomized mice could provide a valuable/more direct hint, as to whether this protein indeed comes only mostly from the pituitary gland.*

In our study, we utilized AAV-mediated CRISPR/Cas9 to disrupt Nptx2 expression in the pituitary, achieving effective knockout but not complete abolition due to incomplete viral infection. Still, we observed a significant reduction in serum Nptx2 levels (**S8I and Fig 3J**), highlighting the pituitary's crucial contribution to systemic Nptx2.

Using transgenic mice with *Nptx2* gene knockout in the pituitary for investigating serum Nptx2 during inflammation would be ideal but very time-consuming. The suggested hypophysectomized mice approach presents challenges due to potential tissue inflammation affecting our analysis, so we decided not to pursue it. As advised by Reviewer 2, we conducted a new experiment showing increased *Nptx2* expression in AtT-20 cells during inflammation (see **Figure R15** below). Our experiments provide valuable data on the pituitary's role in serum Nptx2 levels during inflammation, as discussed in the revised manuscript.

*16.) Was serum Nptx2 concentration also elevated after TNF or Poly I: C injection?*

Our data indicated that serum Nptx2 concentration was not elevated after TNF or Poly (I:C) injection (**Figure R14**).

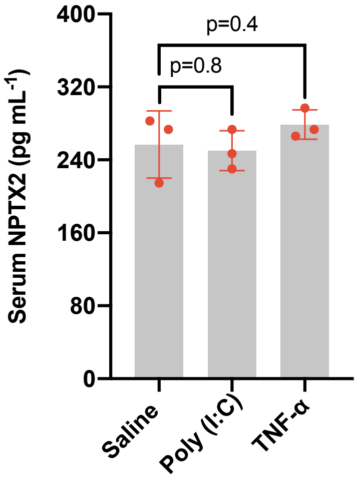


**Figure R14. (A) Serum concentration of Nptx2 from mice treated with 10 mg/kg Poly (I:C) or 0.5 mg/kg TNF-α for 6 hours (n=3).** Statistical significance was determined by two-tailed Student's t-test.

*17.) Of interest, do AtT20 cells also express Nptx2 and can elevate it after LPS or TNF treatment? That would be an easier experiment to do instead of hypophysectomy.*

We acknowledge the practicality of investigating Nptx2 expression in AtT-20 cells in comparison to hypophysectomy. Treating AtT-20 cells with serum from LPS-challenged mice or IL-6 resulted in a notable elevation of *Nptx2* expression (**Figure R15**). These findings, depicted in our revised manuscript (**S8J and S8K Fig**), serve to strengthen our discussion regarding Nptx2's role in inflammation. We extend our gratitude to Reviewer 2 for this valuable suggestion, which has broadened the study's reach and furnished supplementary supportive data.

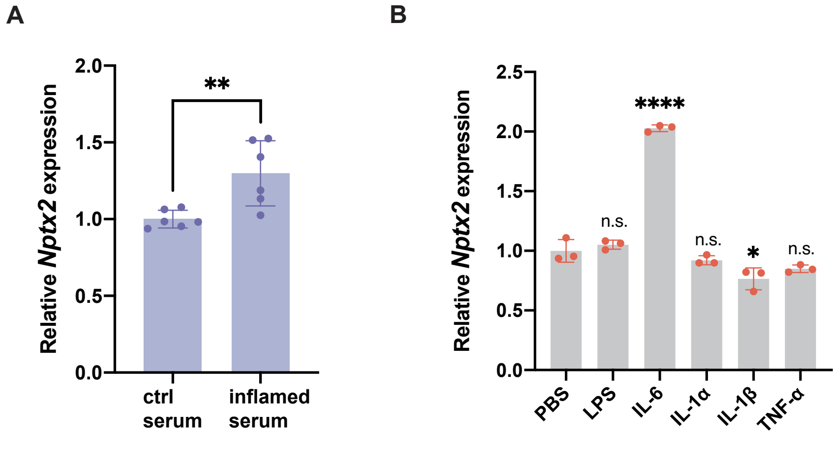


**Figure R15. (A) qPCR analysis of *Nptx2* in AtT-20 cells after treatment with control or inflamed serum for 6 hours (n = 6 replicates). (B) qPCR analysis of *Nptx2* in AtT-20 cells after treatment with PBS, 200 ng/mL LPS, or 100 ng/mL cytokines for 6 hours (n = 3 replicates).** Statistical significance was determined by two-tailed Student's t-test. *p<0.05, **p<0.01, ****p<0.0001.

*18.) I am quite confused why Nptx2 levels were connected to PBMC composition and not for example with pituitary gland damage. Or the nerve synapses in the pituitary.*

We appreciate the chance to elucidate our approach. The pituitary gland, both an endocrine hub and immune contributor, inspired our hypothesis that pituitary HPCs might release factors beyond conventional hormones to influence inflammation during immune challenges. Building on our observation of increased *Nptx2* expression during inflammation and the latest study emphasizing Nptx2's role in modulating complement activity and immune signaling[11], we investigated its potential influence on the immune system. Our focus centered on the blood neutrophil-to-lymphocyte ratio (NLR), a marker for inflammation and immune equilibrium [12, 13].

While considering pituitary damage or nerve synapses is valid, our approach was tailored to prioritize exploring Nptx2's impact on immune responses through a readily measurable parameter. Our examination of how Nptx2 might affect NLR offers a fresh perspective on pituitary modulation of the immune system via non-canonical hormones.

11. Zhou J, Wade SD, Graykowski D, Xiao MF, Zhao B, Giannini LAA, et al. The neuronal pentraxin Nptx2 regulates complement activity and restrains microglia-mediated synapse loss in neurodegeneration. Sci Transl Med. 2023;15(689):eadf0141. Epub 20230329. doi: 10.1126/scitranslmed.adf0141. PubMed PMID: 36989373.

12. Imtiaz F, Shafique K, Mirza SS, Ayoob Z, Vart P, Rao S. Neutrophil lymphocyte ratio as a measure of systemic inflammation in prevalent chronic diseases in Asian population. Int Arch Med. 2012;5(1):2. Epub 2012/01/28. doi: 10.1186/1755-7682-5-2. PubMed PMID: 22281066; PubMed Central PMCID: PMCPMC3277482.

13. Liu CC, Ko HJ, Liu WS, Hung CL, Hu KC, Yu LY, et al. Neutrophil-to-lymphocyte ratio as a predictive marker of metabolic syndrome. Medicine (Baltimore). 2019;98(43):e17537. Epub 2019/10/28. doi: 10.1097/MD.0000000000017537. PubMed PMID: 31651856; PubMed Central PMCID: PMCPMC6824790.

*19.) Alternatively, I would definitely expect some connection with GC effects. It is also weird that rNptx2 alone leads to a decrease in T cells % of PBMC whereas LPS did not (Fig.3I). Moreover, it should be clarified whether injection of rNptx2 in fact modulates GC-exerted effects on PBMC composition. Did rNptx2 injection alter GC levels in LPS-treated mice?*

In response to Reviewer 2's suggestion, we explored the impact of rNptx2 injection on serum glucocorticoid (GC) levels. Our data reveals a decreasing trend in GC levels of LPS-treated mice, although not statistically significant (**Figure R16**). This implies that Nptx2's effect on GC levels is not robust. It's important to acknowledge that the extensive inflammatory responses triggered by LPS treatment create a milieu of pro-inflammatory cytokines, potentially resulting in diverse effects of Nptx2 on PBMCs by modulating C1q complement activity. As we move forward, future studies may provide a more nuanced comprehension of Nptx2's influence on the immune system and pituitary function.

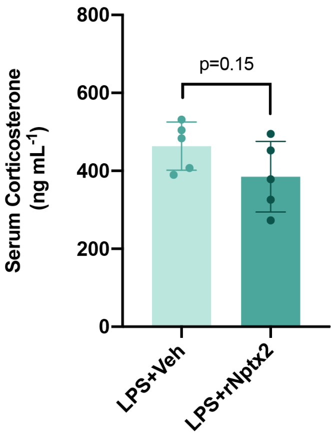


**Figure R16. Serum concentrations of corticosterone.** Mice were pretreated with rNptx2, followed by 0.5 mg/kg LPS for 4 hours. (n = 5 mice). Statistical significance was determined by two-tailed Student's t-test.

*20.) To my opinion the CellChat results and overexpression of Ccl2 does not bring anything to the manuscript except of its title. Chemokines are known to be secreted from different inflamed tissues and they would attract specific immune cells.*

We appreciate the known role of chemokines in attracting immune cells from inflamed tissues. However, our study sought to explore three specific questions about pituitary HPCs: their responses to inflammation, their secretion of non-traditional hormones, and their connections with the immune system. Through CellChat analysis, we discovered extensive crosstalk between HPCs in the pituitary and immune cells in the periphery. This suggests that pituitary HPCs not only regulate the physiological and pathological processes of the body through hormone release but also upregulate the levels of chemokines during inflammation. Likely through chemokine signaling, they engage in direct communication with immune cells, thereby exerting regulatory effects on the body. This finding extends our understanding of pituitary HPC functions beyond the traditional hormone release and underscores their significant role in immune regulation during inflammation.

*21.) The last part of the manuscript (macrophages promote ACTH release under inflammation) is the third independent part of the manuscript (meaning it has been partially known before, and the experiments presented in the Fig. 5 just nicely support it. Those results can be used for an independent manuscript as they have little connection with previous 2 individual parts of the manuscript.*

We thank Reviewer 2 for the suggestion of preparing a separate manuscript based on the third part of the manuscript. Here, we addressed three interconnected questions: 1) How different pituitary HPCs respond to inflammatory stimuli; 2) Whether pituitary HPCs can secrete additional factors other than traditional hormones; and 3) The broad connections between the pituitary and the immune system based on pituitary-secreted factors. **Figures 3** and **4** explored how HPCs influence the composition of immune cells through factor release under inflammatory conditions. In **Figure 5**, we investigated how immune cells within the pituitary

impact hormone secretion, thereby regulating the overall immune response. While these findings can potentially lead to an independent manuscript, our aim as a Resource paper was to provide insights within the article's length limitations. We agree that further molecular and cellular mechanism exploration can be pursued in future studies.

*22.) Line 62: sentence is not clear. Especially considering "mechanisms of pituitary HPC"*

We thank Reviewer 2 for pointing out the clarity issue. In the revised version, we have removed the term "mechanisms of pituitary HPC" from line 62 to ensure accurate and appropriate wording.

*23.) Line 63-4: Unclear Aim. There is a direct immune-HPC interactions mentioned as an aim of study. Which experiments performed in this manuscript had actually addressed it?*

We appreciate Reviewer 2's concern about the clarity of our manuscript's aim. We have now refined our description to avoid ambiguity.

The term "direct immune-HPC interactions" in our original manuscript mainly referred to the reciprocal influence between pituitary HPCs and immune cells under the influence of chemical factors, as demonstrated in experiments such as Transwell assays (**Figure 4E**). Additionally, we conducted supplementary experiments involving the co-culturing of pituitary cells with immune cells *in vitro* and the examination of the impact of macrophages on pituitary hormone secretion *in vivo* (**Figure 5**). These findings suggested the possibility of direct interactions between pituitary cells and immune cells, without mediation by other cell types.

To provide a more precise and unambiguous description, we have revised the wording in lines 63-64 of our revised manuscript as follows: “Lastly, it remains unclear how direct pituitary-immune communication impacts pituitary physiology”. By making this modification, we hope to convey our study's objectives more clearly and provide a better understanding of the significance of our research in unraveling the complexities of immune-HPC interactions in the pituitary gland.

## Reviewer 3

*1.) Overall the organization of the manuscript make is sometimes hard to follow. Particularly, entire subparts of the manuscript are referring entirely to supplementary figures (Supp Fig1, Supp Fig6). Given the importance of the finding discussed in those figures, they should be brought up into the main figures.*

We greatly value Reviewer 3's thoughtful evaluation and valuable suggestions concerning the organization of our manuscript. In response to these insightful comments, we have made necessary revisions to enhance the clarity and flow of the manuscript.

Regarding **S1 Fig**, which contains bulk RNA sequencing data on the pituitary's transcriptional responses to inflammatory challenges, we acknowledge its importance in providing an overview of the information. To ensure that key data are prominently presented, we have now incorporated the essential findings from **S1 Fig** into **Figure 1** of the revised manuscript. This will allow readers to grasp the initial support provided at the population level before delving into the analysis of single-cell RNAseq results with cell type-specificity.

Regarding **S6 Fig**, which reports data on additional peptide-encoding genes in the pituitary gland responding to inflammation, we recognize its relevance in supporting the understanding of the pituitary's secretion abilities during inflammation. However, we also understand that the main conclusions of our study are encapsulated in **Figure 3A-D**. To maintain the primary focus on these key conclusions, we have retained the supporting results in supplementary figures, ensuring that the overall narrative remains coherent.

With these revisions and clarifications, we believe that the key data are now effectively highlighted in the main figures, and the arrangement of supplementary figures supports the primary storyline. We hope that these improvements address Reviewer 3's concerns and contribute to a better understanding and appreciation of our research.

*2.) The authors demonstrate that chemokines are strongly unregulated in HPCs, then move on to Nptx2 to come back to chemokines with the last 2 figures. In this reviewer's opinion, the chemokine story should be one part before moving on to the Nptx2 study. The Nptx2 study is in this reviewer's opinion the most innovative piece of the study in this manuscript, yet some of the important data is placed in a supplementary figure. Some figure and paper re-organization would greatly streamline the messages the authors are putting forward.*

We appreciate Reviewer 3 for the insightful suggestions. We agree that the initial arrangement of our story about chemokines and Nptx2 may appear somewhat disjointed. Our initial intention was to convey the potential of pituitary HPCs to release non-hormone bioactive substances in response to inflammatory challenges when discussing chemokines. Subsequently, we aimed to describe the involvement of pituitary HPCs in systemic immune regulation through the release of non-chemokines.

In our revised manuscript, we addressed this concern by reorganizing the presentation of the chemokine and Nptx2 parts to enhance the continuity and readability of the main storyline. Specifically, we rewrote the first few sentence of the section titled “Nptx2 is a corticotrope-specific factor that modulates systemic inflammation” and we added the figure caption (**Figure**

**3B** and **S7D Fig**) after the first sentence of the section titled "Crosstalks between the pituitary and immune cells during systemic inflammation."

Regarding the Nptx2 study, we acknowledge its significance as one of the most innovative pieces in our manuscript. We understand that some of the important data related to Nptx2 is mainly placed in the supplementary figure in our original manuscript. In our revised manuscript, we adjusted the content arrangement to give proper emphasis to the Nptx2 study. This helps ensure that relevant data and findings related to Nptx2 are presented in a more prominent manner, providing readers with a comprehensive understanding of its significance in the context of our research.

Again, we are grateful to Reviewer 3 for the valuable feedback, which has inspired us to streamline the organization of our paper to better convey our messages.

*3.) In the last 2 figures, the authors aim that "inflamed" HPC produce chemokines to recruit immune cells that then would act on the HPC to produce more hormones. The data as currently presented is circumstantial and the experimental design has some flaws that hinder proper interpretation of the results.*

We appreciate Reviewer 3 for the insightful comments regarding the interpretation of our results and the experimental design. In response, we have made several improvements to clarify and validate our findings.

In our study, we observed an upregulation of myeloid-chemokine pathways at the RNA level in the pituitary gland in response to immune challenges. Interestingly, these upregulated pathways were also evident in hormone-producing cells (HPCs) based on scRNA sequencing results, suggesting that the pituitary gland can produce more than traditional hormones during inflammation. To investigate intercellular communication networks between the pituitary and immune system, we employed CellChat analysis on single-cell RNA-sequencing data, revealing strong connections between HPCs and immune cells. For instance, corticotropes and immune cells exhibited increased activity in the CCL and CXCL signaling pathways.

To validate these observations, we conducted *in vitro* experiments using AtT-20 cells, a well-established ACTH-producing corticotrope cell line, along with bone marrow-derived macrophages (BMDMs), primary monocytes, and primary neutrophils to simulate *in vivo* interactions. qPCR analysis demonstrated that immune stimuli led to increased expression of chemokine genes in AtT-20 cells, and "inflamed" AtT-20 cells attracted BMDMs, neutrophils, and monocytes in Transwell assays. These results supported the notion that "inflamed" HPCs produce chemokines to recruit immune cells.

Furthermore, our cell composition analysis in the pituitary, using FACS after fully perfusing the blood, revealed an elevation in the number of macrophages and neutrophils in the pituitary tissue, indicating that immune cells can be attracted to the pituitary. These findings provided further evidence supporting the idea that HPCs may play a specific role in immune cell recruitment during inflammation.

To explore the functions of infiltrated immune cells, we conducted *in vitro* co-culturing experiments of AtT-20 cells with BMDMs. Our results showed that "inflamed" BMDMs enhanced the release of ACTH from AtT-20 cells. Additionally, *in vivo* depletion of macrophages within the pituitary during inflammation led to a decrease in serum ACTH levels,

while overexpression of CCL2 in the pituitary, which recruits macrophages, resulted in an increase in serum ACTH levels.

While we acknowledge that our experimental design may not be exhaustive, we believe that our data strongly suggest that "inflamed" HPCs produce chemokines to recruit immune cells that then act on the HPC to produce more hormones. We hope that this Resource study provides a strong foundation for further in-depth investigations in future mechanistic studies.

*4.) In Figure 4, the authors show an upregulation of chemokines by HPC upon inflammation that is particularly linked to neutrophil attraction. The authors go on to try to demonstrate immune cell recruitment into the pituitary gland upon LPS challenge. The gating strategy used by the author to discriminate resident macrophages from recruited is based on CD11b expression levels. This reviewer questions the validity of this approach. Other markers should be used to validate that this strategy discriminates between microglia and infiltrating macrophages. The gating strategy and markers to identify neutrophils should also be provided.*

We are grateful to Reviewer 3 for the important technical concerns. In response to this feedback, we have thoroughly revised our manuscript and implemented gating strategies and markers to accurately identify resident macrophages/microglia, recruited macrophages, and neutrophils.

For neutrophil identification, we utilized $CD45^{+}CD11b^{+}Ly6G^{+}$ as the markers, as outlined in the detailed analysis strategy provided in the revised manuscript. To address the concern regarding the macrophage gating strategy, we have made adjustments based on Reviewer 3's suggestion. We use $CD45^{lo}CD11b^{+}$ as the marker for microglia and $CD45^{hi}CD11b^{+}$ as the marker for recruited macrophages [14] (**Figure R17**).

Specifically, we acknowledge that the composition of macrophages and microglia in the pituitary is complex. There are $CX3CR1^{+}TMEM119^{+}$ microglia (indicated by white arrows) mainly located in the posterior and intermedia lobes [15] and $CX3CR1^{+}TMEM119^{-}$ macrophages (indicated by yellow arrows) mainly located in the anterior lobe of the pituitary (**Figure R18**). Therefore, distinguishing pituitary IBA1-positive cells as resident microglia or recruited macrophages during inflammation is not straightforward. In our original manuscript, we used a gating strategy based on yolk sac-derived macrophage/microglia marker ($F4/80^{hi}CD11b^{lo}$) and monocyte-derived cells marker ($F4/80^{lo}CD11b^{hi}$) to distinguish resident and recruited microglia/macrophages [16-19] (which shown in **S9C Fig** in the revised manuscript). Importantly, we have verified that the observed trends in both gating strategies remain consistent (see **Figure R19** below and **Figure 4F**), supporting the validity and reliability of our findings. Based on the above analysis, we believe that using F4/80 and CD11b as markers to distinguish resident and recruited microglia/macrophages is more appropriate.

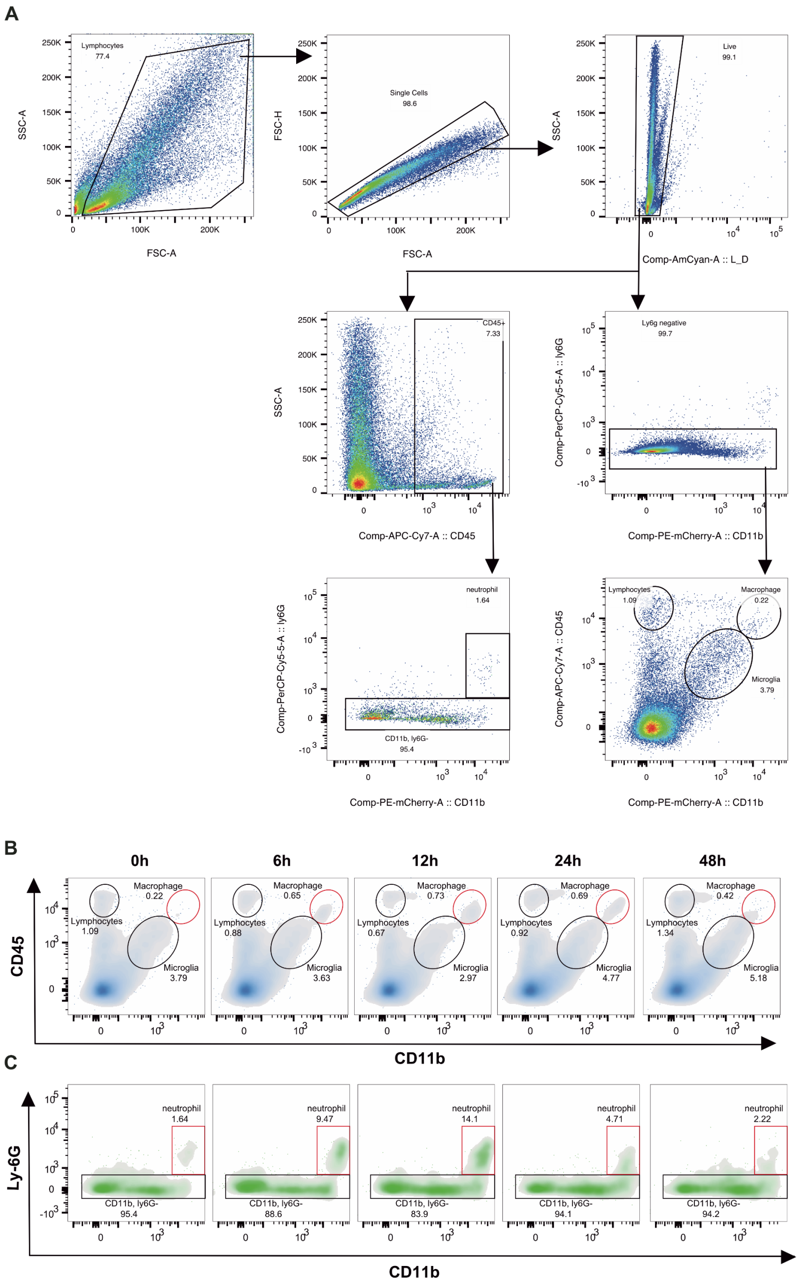


**Figure R17. (A) Gating strategy for neutrophil and macrophage. (B) FACS analysis for resident microglia (black circle) and recruited macrophages (red circle) among live cells in the pituitary from mice treated with LPS at different times. (C) FACS analysis for neutrophils among $CD45^+$ cells in the pituitary from mice treated with LPS at different times.**

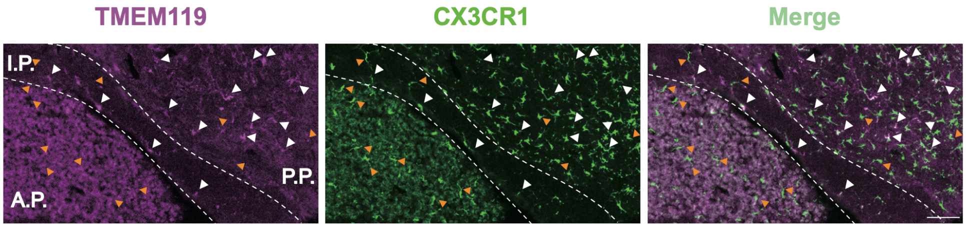


**Figure R18. Representative images showing TMEM119 (purple) staining in the pituitary from CX3CR1-GFP mouse.** A.P., anterior pituitary; I.P., intermediate lobe of the pituitary; P.P., posterior pituitary. White arrows indicate $CX3CR1^{+}TMEM119^{+}$ microglia, and yellow arrows indicate $CX3CR1^{+}TMEM119^{-}$ macrophage. Scale bar, 100 µm.

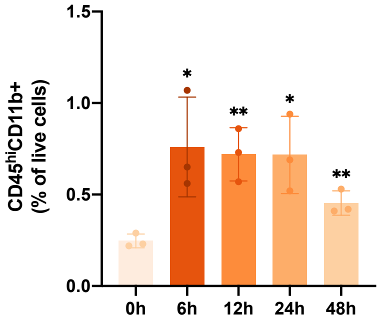


**Figure R19. Flow cytometry analysis of $CD45^{hi}CD11b^{+}$ macrophages in the pituitary from mice treated with saline or LPS (0.5 mg/kg; n=3 mice).** All data represent means±s.e.m. Statistical significance was determined by two-tailed Student's t-test, *p<0.05, **p<0.01.


14. Ford AL, Goodsall AL, Hickey WF, Sedgwick JD. Normal adult ramified microglia separated from other central nervous system macrophages by flow cytometric sorting. Phenotypic differences defined and direct ex vivo antigen presentation to myelin basic protein-reactive CD4+ T cells compared. J Immunol. 1995;154(9):4309-21. PubMed PMID: 7722289.

15. Bennett ML, Bennett FC, Liddelow SA, Ajami B, Zamanian JL, Fernhoff NB, et al. New tools for studying microglia in the mouse and human CNS. Proc Natl Acad Sci U S A. 2016;113(12):E1738-46. Epub 20160216. doi: 10.1073/pnas.1525528113. PubMed PMID: 26884166; PubMed Central PMCID: PMCPMC4812770.

16. Ginhoux F, Guilliams M. Tissue-Resident Macrophage Ontogeny and Homeostasis. Immunity. 2016;44(3):439-49. doi: 10.1016/j.immuni.2016.02.024. PubMed PMID: 26982352.

17. Schulz C, Gomez Perdiguero E, Chorro L, Szabo-Rogers H, Cagnard N, Kierdorf K, et al. A lineage of myeloid cells independent of Myb and hematopoietic stem cells. Science. 2012;336(6077):86-90. Epub 20120322. doi: 10.1126/science.1219179. PubMed PMID: 22442384.

18. Itoh M, Suganami T, Kato H, Kanai S, Shirakawa I, Sakai T, et al. CD11c+ resident macrophages drive hepatocyte death-triggered liver fibrosis in a murine model of nonalcoholic steatohepatitis. JCI Insight. 2017;2(22). Epub 20171116. doi: 10.1172/jci.insight.92902. PubMed PMID: 29202448; PubMed Central PMCID: PMCPMC5752377.

19. Gupta P, Lai SM, Sheng J, Tetlak P, Balachander A, Claser C, et al. Tissue-Resident CD169(+) Macrophages Form a Crucial Front Line against Plasmodium Infection. Cell Rep. 2016;16(6):1749-61. Epub 20160728. doi: 10.1016/j.celrep.2016.07.010. PubMed PMID: 27477286.

*5.) The authors refer to M1 and M2 markers. The macrophage field has now moving away from this nomenclature that fails to illustrate the actual dynamic of macrophage phenotype. The author should refrain from using these terms.*

Thank you for bringing up the issue of using M1 and M2 terminologies for macrophage classification. In our revised manuscript, we have made the necessary changes and replaced these terms with more specific and informative cell surface markers. For our FACS study, we utilized $CD206^+CD11c^-$ to characterize M2-like macrophages and $CD206^-CD11c^+$ to designate M1-like macrophages. At the transcriptional level, we used $Mrc1^+$ and $CD301^+$ to delineate M2-like macrophages.

We believe that adopting these updated markers provides a clearer representation of the macrophage subsets we analyzed and aligns with the latest advancements in macrophage research.

*6.) The authors should validate the immune infiltration into the pituitary using histology to really demonstrate the intraparenchymal infiltration upon LPS challenge.*

We appreciate the valuable suggestion from Reviewer 3 regarding the validation of immune infiltration in the pituitary. In response, we have added histology data showing the spatial distribution of neutrophils and macrophages in the revised manuscript. The histology data of neutrophils have been illustrated in **Figure R1**, and the spatial distribution of macrophages is displayed in **Figure R20** below. Our results indicate that under physiological conditions, there is a substantial number of resident macrophages within the pituitary. However, during inflammation, we observed an increased co-localization of macrophages with vascular cells, and concomitantly, the immunogenicity of macrophages was enhanced, leading to substantial morphological changes, characterized by enlarged cell bodies and reduced processes.

By incorporating these histology data, we have provided a more comprehensive validation of immune infiltration in the pituitary gland upon LPS challenge. These findings further strengthen the support for our study's central theme and contribute to a better understanding of the dynamic changes in immune cell distribution and morphology in response to inflammation.

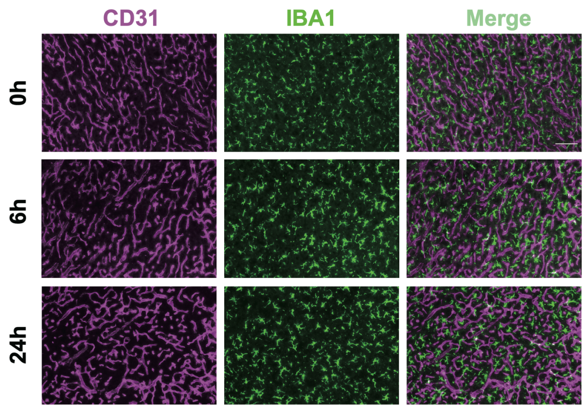

**Figure R20. Representative images showing CD31 (the marker of endothelial cells) and IBA1 (the marker of macrophage) expression in the pituitary from mice treated with saline and 0.5 mg/kg LPS.** Scale bar, 100 μm.

*7.) In the last figure, the authors use local clodronate liposomes to assess how "macrophages" recruitment impact ACTH production upon LPS challenge. As denoted in Figure 5C, the approach depletes infiltrating macrophages (along with other peripheral immune cells) but also microglia. This caveat should be disclosed and discussed in the manuscript as it can be a strong confounding factor in the interpretation of their results. Does clodronate liposomes affects ACTH production at baseline? If so this would suggest that microglia are essential for ACTH production by HPC.*

We thank Reviewer 3 for raising a crucial concern about our use of local clodronate liposomes in the study. As Reviewer 3 pointed out, this approach not only depletes recruited macrophages but also affects local macrophages and microglia. Following this advice, we have disclosed and discussed this issue in the revised manuscript.

Our new findings (**Figure R20**) indicate that under inflammatory conditions, macrophages in the pituitary undergo significant morphological changes, and their overall immunogenicity strengthens. In this state, inflammatory macrophages may impact ACTH release from corticotropes through cytokine release or physical interaction with corticotropes. Although we did not strictly differentiate between affected macrophage types, including resident microglia/macrophages and recruited macrophages, the overall macrophage population in the pituitary is likely influenced by local clodronate liposomes under inflammatory conditions.

Regarding Reviewer 3's question about the impact of macrophages on baseline ACTH production, we conducted additional investigations to address this. Our results (**Figure R21**) demonstrate that liposome-clodronate treatment does not affect baseline ACTH production. Therefore, it suggests that macrophages do not significantly regulate ACTH production under physiological conditions.

In conclusion, our new findings contribute to a more comprehensive understanding of macrophages' role in ACTH production under inflammatory conditions. By disclosing and discussing these important aspects in the revised manuscript, we aim to provide readers with a better grasp of the potential implications and limitations of our experimental approach.

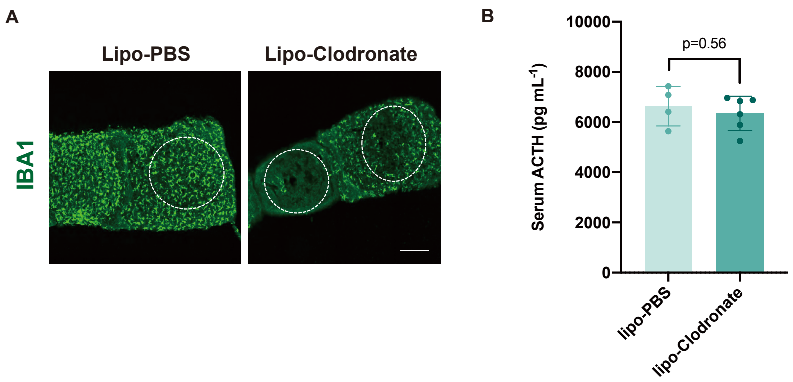

**Figure R21. (A) Representative images showing IBA1 (the marker of macrophage) expression in the pituitary from mice that received liposome-PBS (left) or liposome-Clodronate (right) directly to the pituitary for 24 hours.** Scale bar, 250 μm. **(B) Serum concentrations of ACTH in mice treated with liposome-PBS or liposome-Clodronate (n= 4 and 6 mice, respectively).** Statistical significance was determined by a two-tailed Student's t-test.

*8.) Figure 4 emphasize how neutrophils seem to be a primary target, but the author moves on to macrophages in Figure 5. While challenging, depletion of neutrophils has been demonstrated by multiple groups. The authors could also modulate neutrophils recruitment by blocking adhesion molecule on the blood vasculature, or block some of the identified chemokines using commercially available blocking antibodies. These experiments, coupled with the CCL2 experiments already presented would greatly strengthen this part of the manuscript.*

We recognize the potential benefits of manipulating neutrophils to investigate their role in-depth. Modulating neutrophil recruitment with blocking antibodies or other methods could strengthen the manuscript, offering a more direct examination of their impact on the pituitary gland. However, achieving local manipulation within the pituitary is crucial for more meaningful insights into neutrophils' specific role. Currently, satisfactory methods for local manipulation have not been identified.

Considering these limitations, we focused our experiments on macrophages, which are well-established and technically feasible to manipulate. By studying immune cells, including macrophages, and their impact on pituitary HPC functionality, we aimed to provide some initial insights into the overall immune response. Given the scope of this manuscript as a Resource paper, the inclusion of macrophage experiments makes some meaningful contributions to understanding immune interactions in the pituitary gland.

While neutrophil manipulation was not explored in this study, we acknowledge the importance of Reviewer 3's suggestion. Future studies can incorporate experiments involving neutrophil recruitment modulation or blocking strategies to further investigate their specific role in pituitary function and the immune response.

*9.) Have the authors look if macrophages/neutrophils recruitment are also the driver for Nptx2 to serve as a negative feedback loop to limit uncontrolled inflammation? Attempt to link the two major parties of this manuscript would be fantastic, although not required to find this manuscript suitable for publication.*

We agree that exploring the potential link between Nptx2 expression and macrophage/neutrophil recruitment in the context of a negative feedback loop to limit uncontrolled inflammation could provide valuable insights. In our revised manuscript, we have included some additional information to address this intriguing idea.

As shown in **Figure R15**, we demonstrated that IL-6, but not several other cytokines, significantly upregulates *Nptx2* expression in AtT-20 cells. As literature reported that macrophages as one of the primary cellular sources of IL-6 in response to immune challenges [19], this information implies that, under inflammatory conditions, the recruitment of macrophages and neutrophils to the pituitary may lead to the secretion of cytokines, such as IL-

6, which potentially act as a trigger for corticotropes to release Nptx2 to regulate inflammatory responses. While we recognize the significance of this potential link, we acknowledge that further in-depth investigations are warranted to fully understand the underlying mechanisms and interactions involved. Due to time limitations, we were unable to conduct a more comprehensive exploration of this topic. However, we believe that our initial findings and proposal open up exciting avenues for future research in this area.

19. Arango Duque G, Descoteaux A. Macrophage cytokines: involvement in immunity and infectious diseases. Front Immunol. 2014;5:491. Epub 20141007. doi: 10.3389/fimmu.2014.00491. PubMed PMID: 25339958; PubMed Central PMCID: PMCPMC4188125.

*10.) The IHC data presented in figure 3 and Supp figure 5 are hard to read, even on a computer. The addition of zoomed in inset would greatly help the reading of these figures.*

We appreciate the valuable feedback from Reviewer 3 concerning the readability of the IHC data in Figure 3 and Supplementary Figure 5. To address the concern, in **Figure 3** we have included zoomed-in insets for the IHC images to provide a more detailed view of the *Nptx2* expression (also see **Figure R13** above). Similarly, we have incorporated zoomed-in insets for the ISH images of *Crhr1* RNA signals in **S8D Fig**. This addition allows for better visualization of the Crhr1 expression patterns (also see **Figure R22** below). We agree that these improvements enhance the readability and interpretation of the IHC data, providing clearer insights into the expression patterns of *Nptx2* and *Crhr1*.

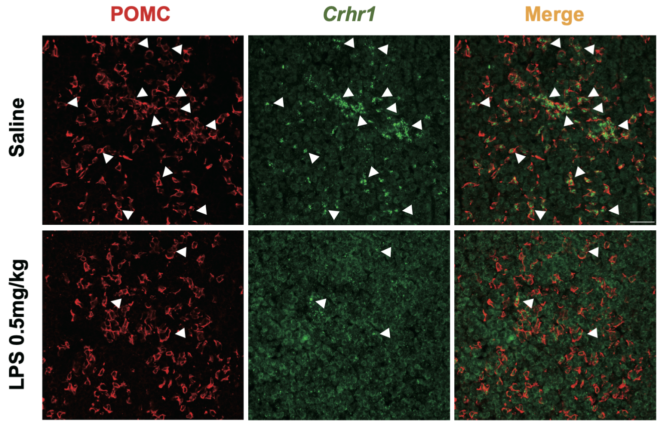


**Figure R22. Representative images showing ISH of *Crhr1* RNA (green) and IF of POMC (red) in the pituitary from mice treated with saline or 0.5 mg/kg LPS for 6 hours.** Arrows indicate cells co-expressing *Crhr1* and POMC. Scale bar, 50 µm.

*11.) In figure S4G, the author should also provide the statistical representation of the pathways to reinforce that these were not randomly chosen and rather where mathematically shown to be important.*

We thank Reviewer 3 for pointing out the need of reinforcing the significance of the chosen pathways in Figure S4G. To comprehensively summarize and categorize the signaling pathways associated with each HPC type during inflammation, we conducted a REVIGO

analysis on both shared and unique Gene Ontology (GO) biological process (BP) cellular signaling pathways for each HPC type within the pituitary (**Figure R23**). In the initial manuscript, we solely presented the summarized pathways post REVIGO analysis, which could potentially lead to confusion. In the revised manuscript (**S5 Fig**), in line with the guidance from Reviewer 3, we have furnished specific signaling pathway entries for the REVIGO analysis of each HPC (presented in **Figure R23**). To facilitate reader access to the REVIGO analysis, we have included the URL (http://revigo.irb.hr), where they can find more comprehensive information regarding the analysis methodology and results.

We believe that this additional information will enhance the clarity and transparency of our results, helping readers to better understand how these pathways were quantitatively determined to be important.

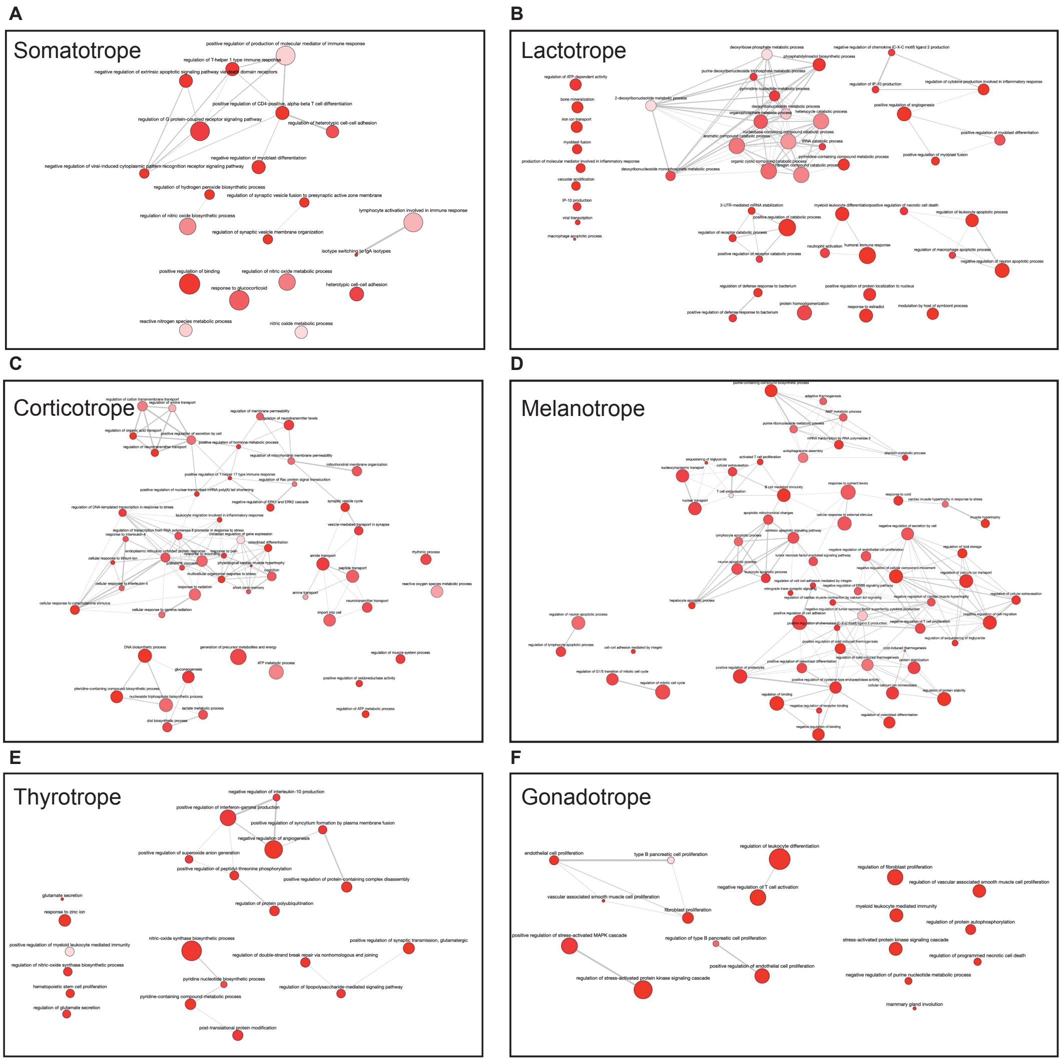


**Figure R23. REVIGO analysis of 6 HPCs.** Bubble color indicates the p-value. Bubble size indicates the frequency of the GO term in the underlying GOA database.

*12.) Figure 1H is extremely hard to read and interpret and seems redundant with Figure 2B. The authors may consider another representation of the results that is easier to read or remove it completely.*

We thank Reviewer 3 for the valuable feedback regarding the readability and potential redundancy of **Figure 1H** in the original manuscript, which was originally intended to provide a more detailed view of the SCENIC analysis presented in **Figures 1F** and **1G** (in the original manuscript) to further support the binary classification of cell states. However, we recognize that the original heatmap might have posed challenges in terms of readability and interpreting the results.

To address this concern, we have made substantial improvements to **Figure 1J** in the revised manuscript. Firstly, we have replaced the original heatmap with a new version (**Figure R24** below). This revised version offers a zoomed-in view that highlights the regulons exhibiting differential activation across HPC cell types or cell states. By focusing on these specific regulons, we aim to enhance the clarity of the results and make them easier to interpret. Secondly, to further streamline the presentation of our results, we have moved the complete heatmap to **S2F Fig**. This relocation allows us to maintain the valuable information while avoiding redundancy.

We believe that these revisions address the concerns raised by the reviewer. We are confident that the updated figures will provide a more accessible representation of our findings.

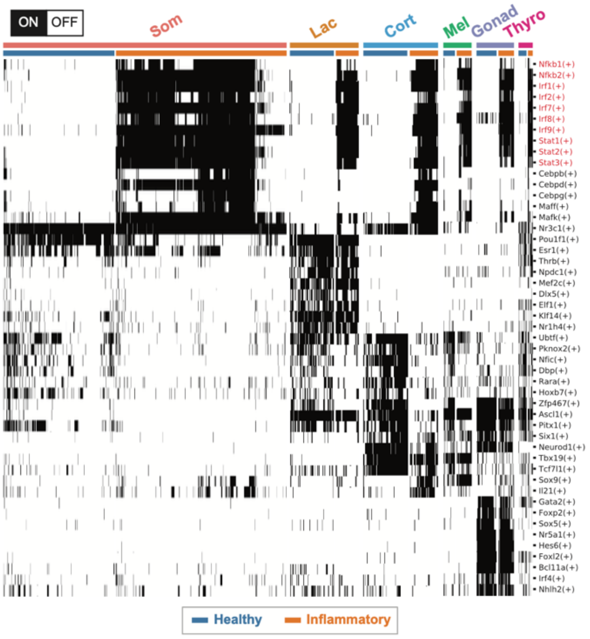


**Figure R24. Heatmap showing the binarized SCENIC regulon activity in HPCs with healthy and inflammatory cell states.**

*13.) On page 4, the authors refer to TNFa injection as a "cytokine storm". I would encourage the authors to avoid using this term as Cytokine Storm is defined by the simultaneous massive upregulation of numerous cytokines.*

Thank you. We have replaced the term “cytokine storm” with “proinflammatory cytokine” in the revised manuscript.
