# Supplementary material for: Single-cell transcriptomic analysis reveals rich pituitary–Immune interactions under systemic inflammation
Source: PLoS Biol. 2023 Dec 18;21(12):e3002403. doi: 10.1371/journal.pbio.3002403 (PMC10727439; doi:10.1371/journal.pbio.3002403)
Supplement: Attachment — Submitted filename: PBIOLOGY-D-23-00680_R3_response to reviewer.docx [file pbio.3002403.s026.docx]

**Point-by-point Response**

We are gratified to learn that both Reviewers 1 and 2 recommend to accept the manuscript for publication. We extend our sincere appreciation for Reviewer 3's overall satisfaction with our response. We have further addressed Reviewer 3's remaining concerns through figure editing and text revisions.

**Reviewer #1:** *The authors have fully addressed all of my concerns.*

We thank Reviewer 1 for his/her valuable support and meticulous review, which significantly contributed to enhancing the quality of this study.

**Reviewer #2:** *I have no further comment.*

Again, we thank Reviewer 2 for his/her excellent comments.

**Reviewer #3:** *First I would like to thank the authors for addressing the majority of my concerns.*

*Some concerns however remain.*

Thank you.

*1.) Some new figures that are provided to the reviewers in the point-by-point section do not appear to have been included in the revised manuscript (R17, R20, R21...)*

We appreciate Reviewer 3's recognition of the data. In response to the feedback, we have incorporated **Figure R17** in the previous rebuttal letter into **S9 Fig**, **R19** into **Figure 4F** and **R21** into **Figure** **5D** in the revised manuscript. It's important to note that **R20** was already included in **S9 Fig** in the previous revision. We apologize for any confusion regarding the labeling in our previous **Point-by-point Response**.

*2.) Regarding figure R21, the description by the authors is suggesting that their interpretation of Iba1 staining in close proximity to blood vasculature demonstrates macrophages infiltration? Multiple reports, including 2* *photon microscope (Davalos et al, 2012) demonstrate that resident microglia will migrate toward blood vessels during inflammation. The use of multiple markers (Iba1, Tmem119, ...) would help the authors differentiate between microglia and infiltrating macrophages.*

We concur that it is important to minimize any potential confusion regarding infiltrating macrophages. We appreciate the Editors' insightful suggestion that we can address the issue by textual revisions. We now use the term "recruited macrophages" in lines 310 (page 13) and 320 (page 14). Furthermore, in line 336 (page 14), we have removed the mention of the potential effect of CCL2 on infiltrating macrophages.
